# Supplementary material for: Genomic heterogeneity of multiple synchronous lung cancer
Source: Nat Commun. 2016 Oct 21;7:13200. doi: 10.1038/ncomms13200 (PMC5078731; doi:10.1038/ncomms13200)
Supplement: Supplementary Information — Supplementary Figures 1 - 8 and Supplementary Tables 1-7 [file ncomms13200-s1.pdf]

Supplementary Figures

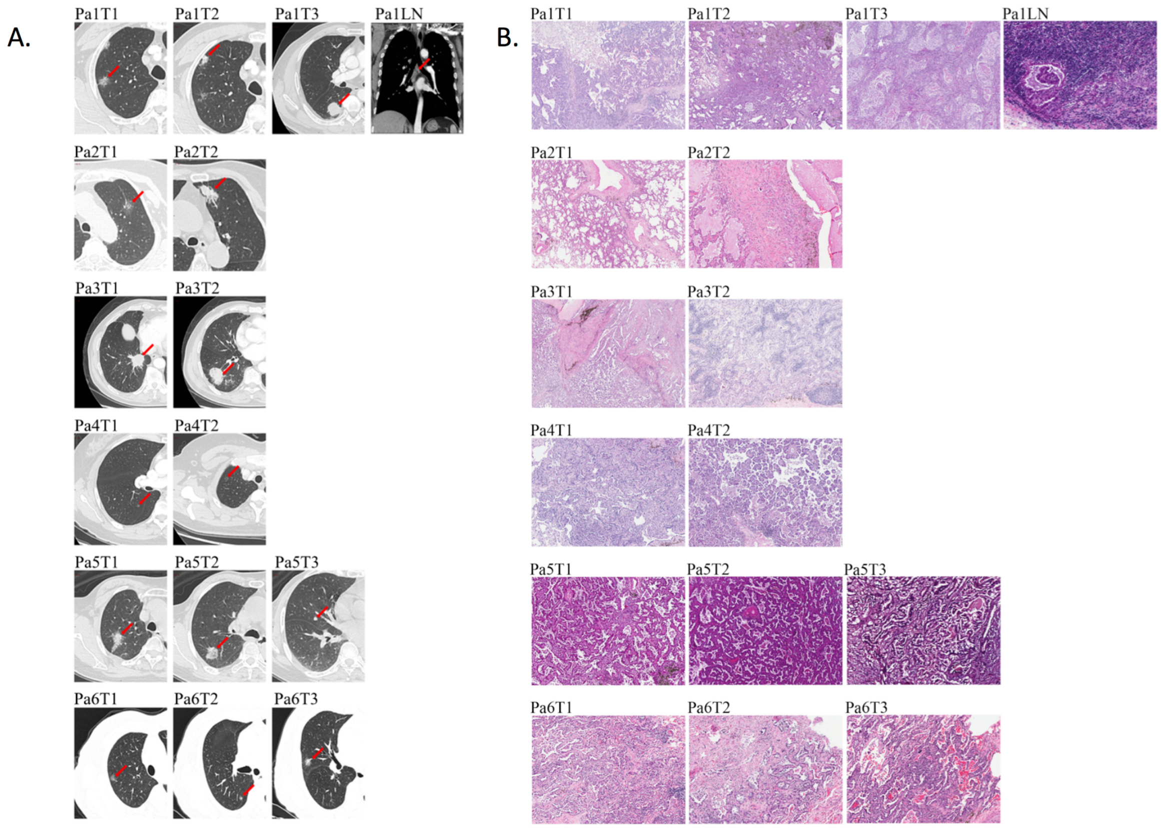

Supplementary Figure 1. Representative computed tomography (A) and hematoxylin-eosin staining (B) images of 16 intra-thoracic MSLC lesions. Pa, patient; T, tumor; LN, lymph node metastasis.

Fig. S2

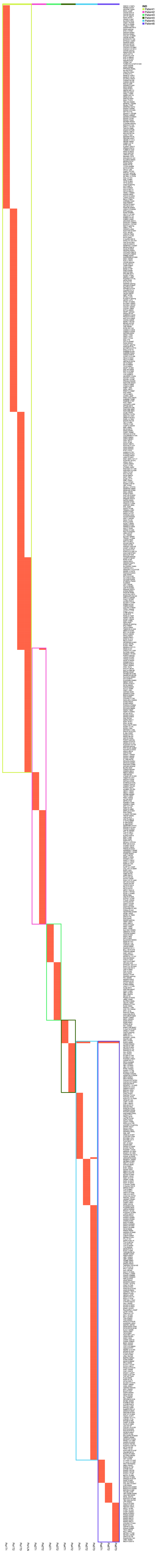
IND
Patient1
Patient2
Patient3
Patient4
Patient5
Patient6
Pa1T1
Pa1T2
Pa1T3
Pa1LN
Pa2T1
Pa2T2
Pa3T1
Pa3T2
Pa4T1
Pa4T2
Pa5T1
Pa5T2
Pa5T3
Pa6T1
Pa6T2
Pa6T3

Supplementary Figure 2. Distribution of validated somatic nonsynonymous coding mutations and splice site mutations in 16 MSLC lesions. Tumors (T) from the same patients (Pa) are grouped by colored rectangles. Mutations are listed on the right. LN, lymph node metastasis.

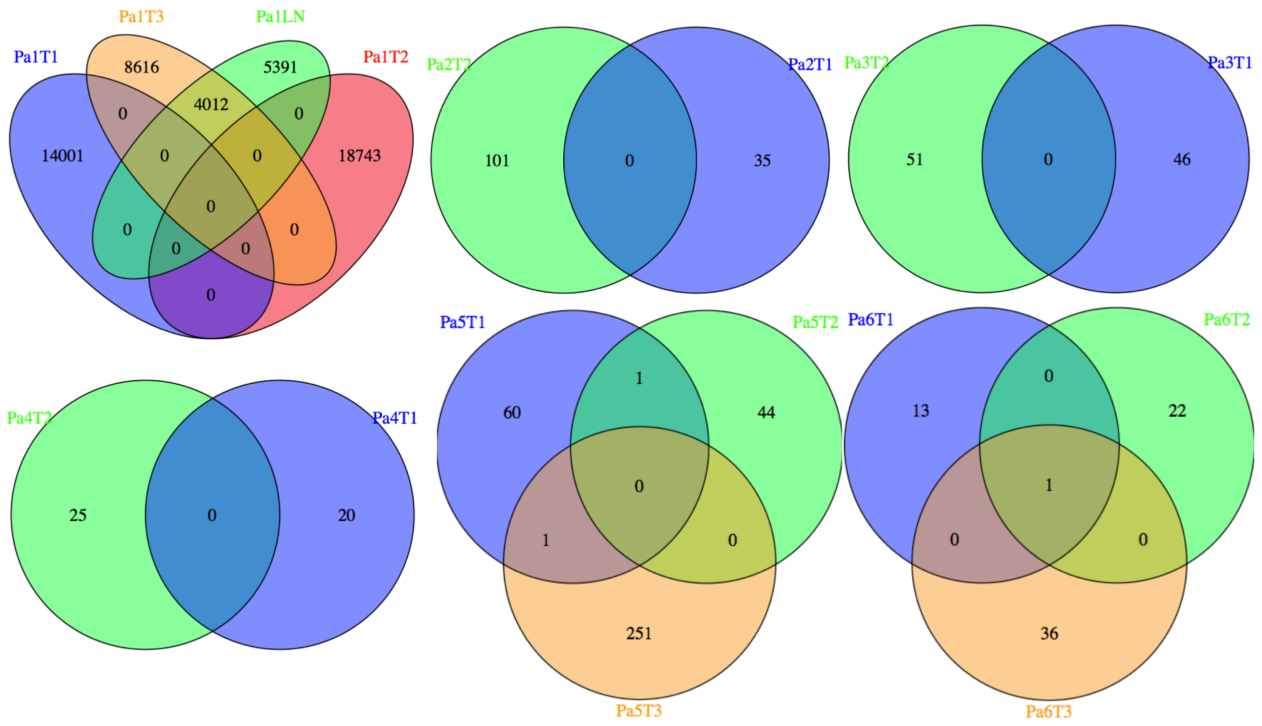

Supplementary Figure 3. Venn diagram illustrating the distributions of validated mutations and all mutations called by both MuTect and VarScan in 16 MSLC lesions. The numbers of mutations identified in only one tumor (T) and the numbers of mutations shared by two or more lesions are indicated. Shared mutations were defined as identical nucleotide substitutions at identical genomic coordinates in different tumors. Pa, patient; LN, lymph node metastasis.

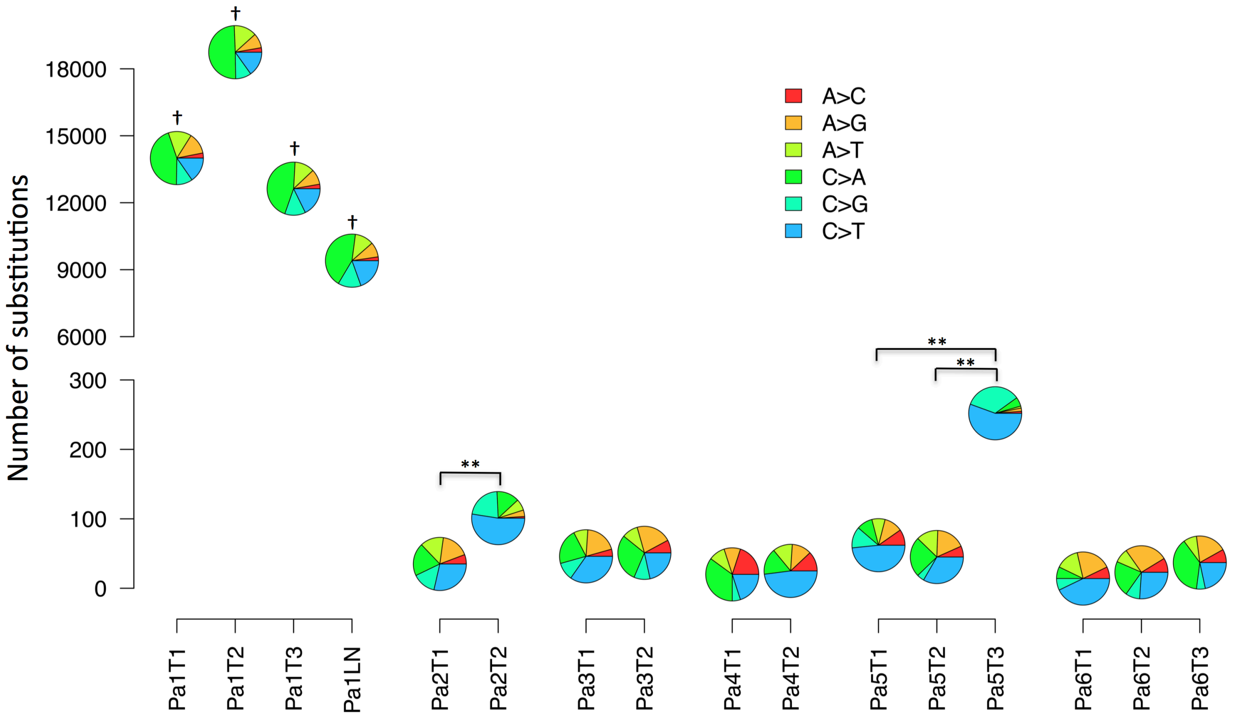

Supplementary Figure 4. Mutation spectra of all validated mutations and mutations called by both MuTect and VarScan in 16 MSLC lesions. †The mutation spectra of the four MSLC lesions of patient 1 (Pa1) were highly correlated ($R > 0.97$ and $p < 0.01$ between any two lesions by the Pearson product-moment correlation analysis) but also significantly different ($p < 0.0005$ between any two lesions by the Fisher exact test). **The mutational spectra of the tumors (T) of patient 2 differed significantly from each other, as did the mutational spectra of the tumors 1 and 3 and tumors 2 and 3 of patient 5 ($p < 0.01$, Fisher exact test). LN, lymph node metastasis.

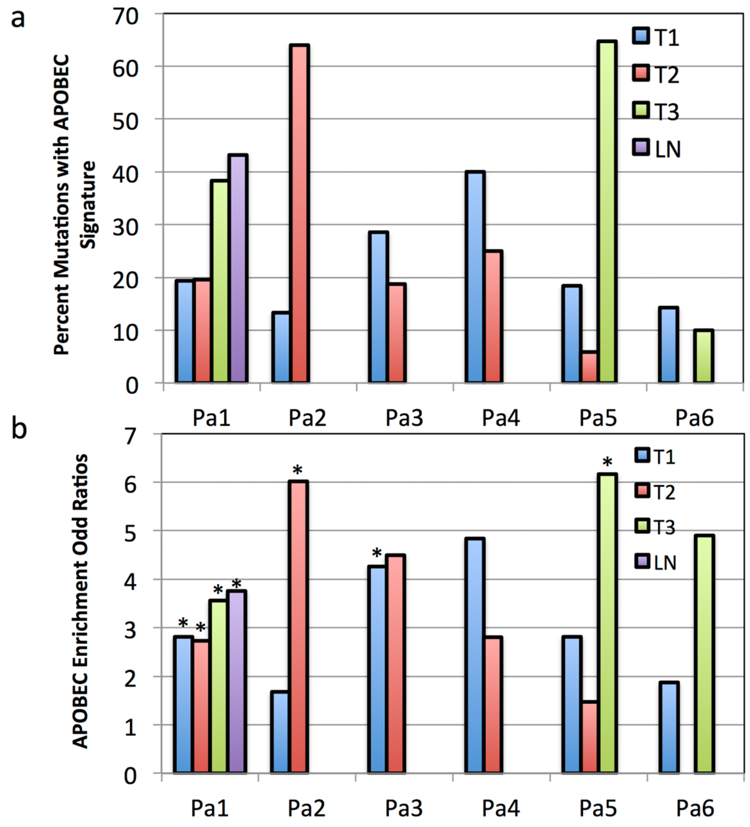

Supplementary Figure 5. APOBEC signatures in 16 MSLC lesions. All validated mutations and mutations called by both MuTect and VarScanwere included in the analysis. (*A*) Percentages of mutations with an APOBEC signature in the 16 MSLC lesions. (*B*) APOBEC enrichment odds ratios for the 16 MSLC lesions. *APOBEC enrichment was statistically significant in seven lesions ($p < 0.05$, Fisher exact test). Pa, patient; T, tumor; LN, lymph node metastasis.

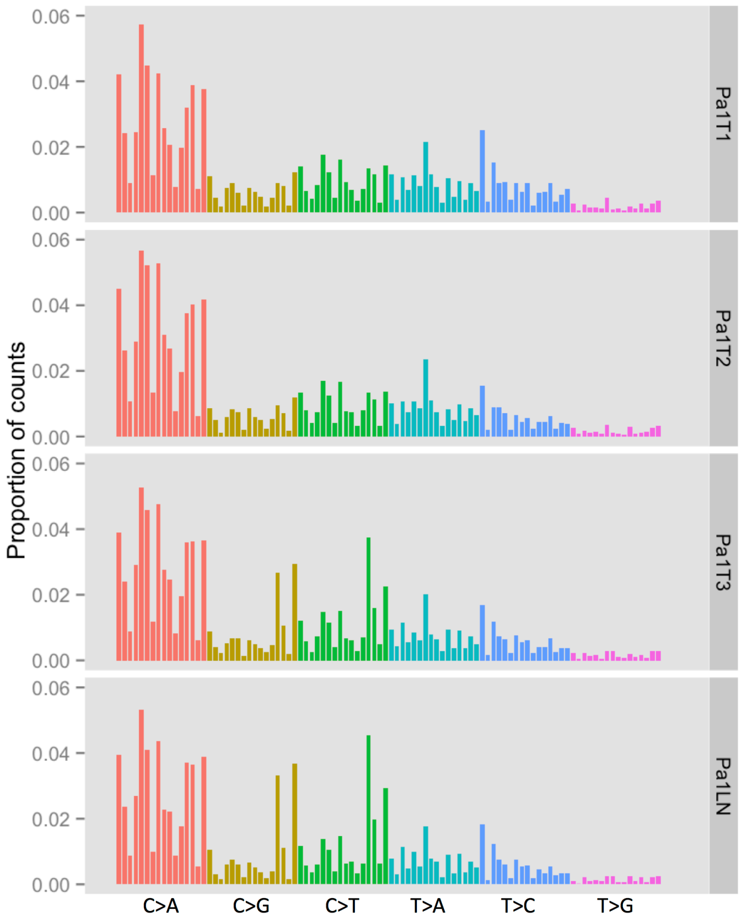

Supplementary Figure 6. Mutation signature analysis of three MSLC tumors (T) and a lymph node metastasis (LN) of patient 1 (Pa1). All validated mutations and mutations called by both MuTect and VarScan were included in the analysis. Nucleotide substitutions in all mutations were grouped into six categories as indicated on the x-axis. In each of the six categories, mutations were then grouped into 16 subcategories according to the bases immediately 5’ and 3’ to each mutated base. The data are the relative frequencies of the six mutation types in the 16 contexts.

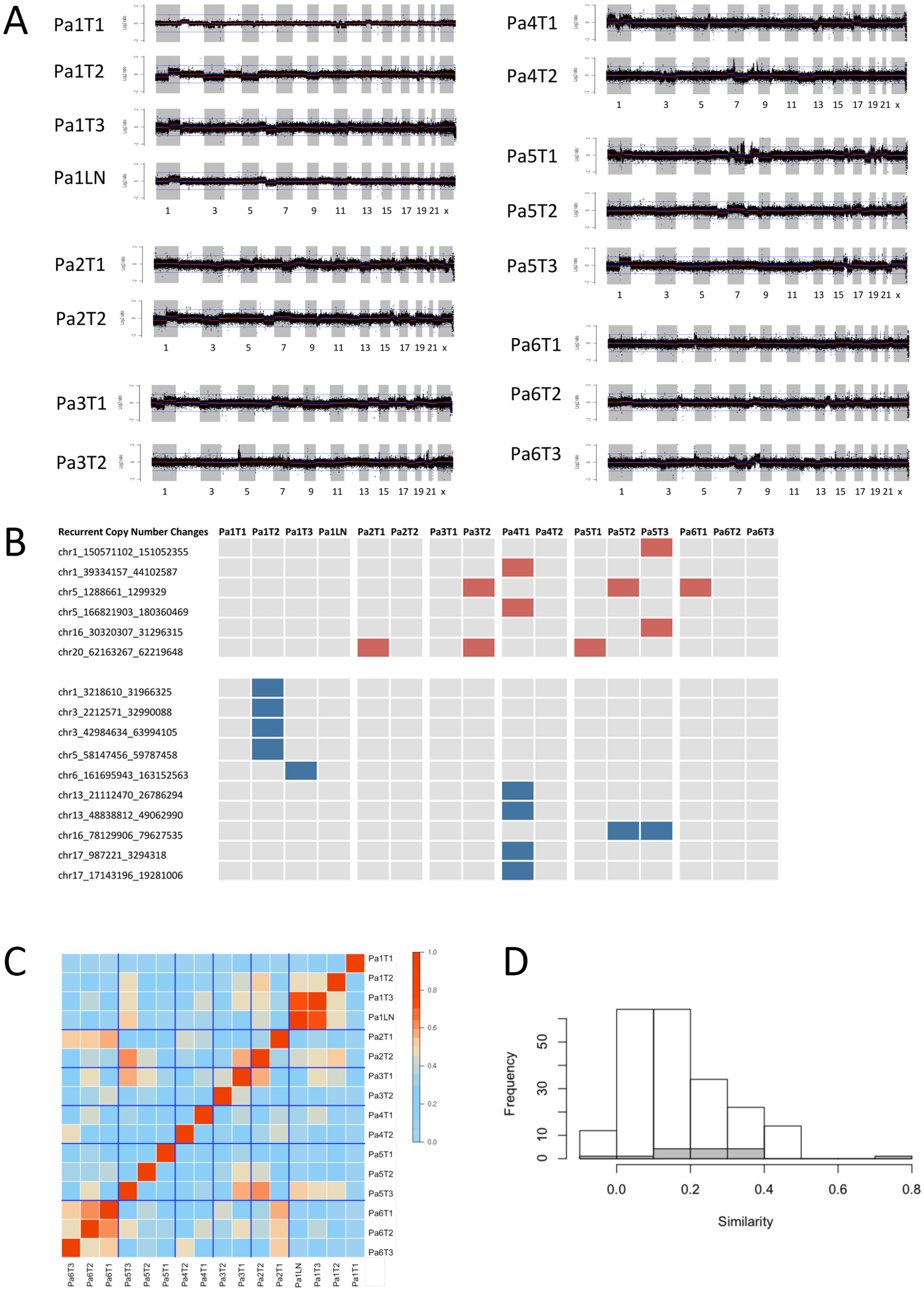

Supplementary Figure 7. (*A*) SCNAs in 16 MSLC lesions identified by microarray-based CGH. The data are $\log_2$ ratios of tumor DNA versus germline DNA array signals.Chromosome numbers are indicated on the x-axis. Pa, patient; T, tumor; LN, lymph node metastasis.(*B*)

Distribution of recurrently gained (in red;$\log_2$ ratio > 0.3) and lost (in blue;$\log_2$ ratio < –0.3) chromosomal (chr) segments (based on TCGA lung adenocarcinoma data) in 16 MSLC lesions. (*C*) Correlation of SCNAs identified by microarray-based CGH among the 16 MSLC lesions. The heatmap represents correlation coefficients of segment $\log_2$ ratios (tumor DNA to germline DNA) for each pair of lesions. (*D*) Likelihood ratios against a background reference distribution. The open bars represent the log likelihood ratios for all comparisons of 16 MSLC lesions (128 pairings), which serve as a reference distribution. The gray shading represents the 22 possible same-patient tumor pairings. Only one pair — tumor 3 and the lymph node metastasis of patient 1—had a higher likelihood than the largest reference comparison, indicating clonality (i.e., the metastasis likely originated from tumor 3). The remaining comparisons were consistent with the reference distribution and were therefore considered to indicate independent multiple primary tumors.

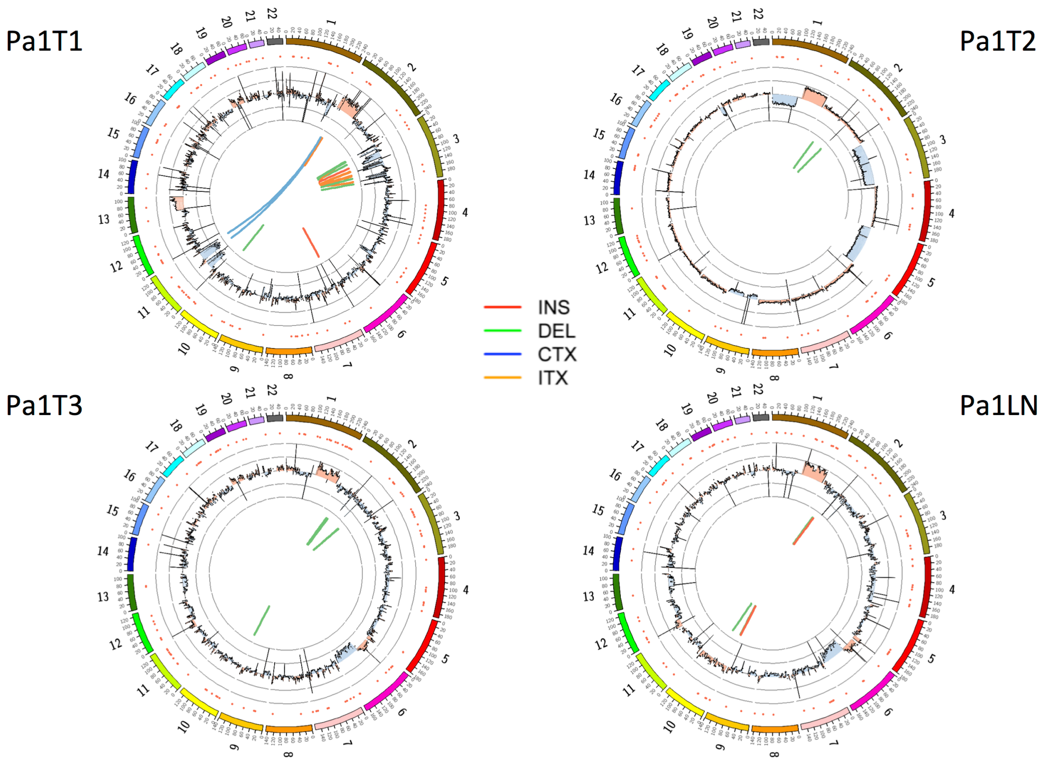

Supplementary Figure 8. Genomic alterations detected by WGS in the four MSLC lesions of patient 1 (Pa1). Chromosomes are indicated on the outer ring. The somatic aberrations shown (from outside in) are validated mutations, copy number alterations (histogram), and validated somatic DNA structural variants. INS, insertion; DEL, deletion; CTX, interchromosomal translocation; ITX, intrachromosome translocation; T, tumor; LN, lymph node metastasis.

Supplementary Table 1. Histomorphological subtypes* and their percentage in the 16 intrathoracic lesions.

Code	LesionID	Location†	Histology‡	Radiology Observation§	Subtype (%)					
					Minimally Invasive Adenocarcinoma	Lepidic Predominant	Acinar	Papillary	Micro-Papillary	Solid
Patient 1	Pa1T1	RUL	ADC	GGO	0	95	5	0	0	0
	Pa1T2	RUL	ADC	Part solid	0	5	10	85	0	0
	Pa1T3	RLL	ADC	Solid	0	0	85	0	5	10
	Pa1LN	LN	ADC		0	0	100	0	0	0
Patient 2	Pa2T1	LUL	ADC	Part solid	0	95	5	0	0	0
	Pa2T2	LUL	ADC	Solid	0	25	75	0	0	0
Patient 3	Pa3T1	RLL	ADC	Solid	0	5	50	25	20	0
	Pa3T2	RLL	ADC	Solid	0	5	80	10	5	0
Patient 4	Pa4T1	RUL	ADC	GGO	0	5	95	0	0	0
	Pa4T2	RUL	ADC	GGO	0	5	0	95	0	0
Patient 5	Pa5T1	RUL	ADC	Solid	0	5	5	90	0	0
	Pa5T2	RUL	ADC	Solid	0	0	5	95	0	0
	Pa5T3	RML	ADC	Solid	0	0	100	0	0	0
Patient 6	Pa6T1	RUL	ADC	Part solid	0	100	0	0	0	0
	Pa6T2	RUL	ADC	Part solid	0	100	0	0	0	0
	Pa6T3	RML	ADC	Part solid	100	0	0	0	0	0

* According to the multidiscipline classification criteria for adenocarcinoma in 2011.

† RUL, right upper lobe; RLL, right lower lobe; LN, Lymph Node; LUL, left upper lobe; RML, right middle lobe

‡ ADC, adenocarcinoma

§ GGO, ground glass opacity

Supplementary Table 2. Summary of somatic alterations detected in tumors from Patient 1 by whole-genome sequencing.

Type of Alterations	Pa1T1	Pa1T2	Pa1T3	Pa1LN
**Single Nucleotide Variants***	26000	26838	28971	29728
Coding	151	154	216	248
Nonsense	6	7	16	18
Missense	110	106	149	166
Synonymous	35	41	51	64
Non-coding	1192	1211	1437	1462
UTR	133	118	181	177
ncRNA	1059	1093	1256	1285
Intronic	6896	6994	8178	8872
Splice site	6	7	4	8
Other	6890	6987	8174	8864
Intergenic	17761	18479	19140	19146
**Small Indels**	1,081	1,306	1,124	1,612
Coding	0	1	0	1
Non-coding	42	39	43	10
Intronic	418	461	441	687
Intergenic	621	805	640	914
**Rearrangements**	19	2	6	7
Intrachromosomal	16	2	6	7
DEL	7	2	5	5
ITX	5	0	0	0
INS	4	0	1	2
Interchromosomal	3	0	0	0

* Single Nucleotide Variants (SNV) called by either Mutect or Varscan.

Supplementary Table 3. Summary of somatic alterations detected in tumors from Patients 2-6 by whole exome sequencing.

Type of Change	Pa2T1	Pa2T2	Pa3T1	Pa3T2	Pa4T1	Pa4T2	Pa5T1	Pa5T2	Pa5T3	Pa6T1	Pa6T2	Pa6T3
**Single Nucleotide Variants***	98	192	109	116	47	79	119	75	431	45	80	99
Coding	45	85	45	58	25	31	51	36	206	23	28	43
Nonsense	1	4	1	2	1	3	0	2	14	1	1	5
Missense	30	64	34	42	19	24	41	28	133	20	21	28
Synonymous	14	17	10	14	5	4	10	6	59	2	6	10
Non-coding	5	20	10	13	8	9	8	6	19	1	6	8
UTR	2	10	10	9	0	6	1	4	8	1	1	2
ncRNA	3	10	0	4	8	3	7	2	11	0	5	6
Intronic	38	68	42	29	10	30	44	24	160	15	34	32
Splice site	0	1	0	1	1	0	1	0	0	0	1	0
Other	38	67	42	28	9	30	43	24	160	15	33	32
Intergenic	10	19	12	16	4	9	16	9	46	6	12	16
**Small InDels**	8	2	12	7	15	5	13	14	19	12	16	15
Coding	1	0	1	0	1	1	1	2	1	0	0	1
Non-coding	0	0	0	0	2	0	2	3	4	0	2	1
Intronic	8	2	11	7	10	3	9	7	13	10	14	11
Intergenic	0	0	0	0	2	1	1	2	1	2	0	2

* Single Nucleotide Variants (SNV) called by either Mutect or Varscan were included.

Supplementary Table 4. Validated Mutations

Tumor ID	Chrom	Position	Gene	cDNA change	AA change	Function
Pa1T1	chr1	24448028	IL22RA1	c.A992T	p.D331V	exonic nonsynonymous SNV
Pa1T1	chr1	26691643	ZNF683	c.G394C	p.D132H	exonic nonsynonymous SNV
Pa1T1	chr1	55119581	HEATR8	c.A982T	p.T328S	exonic nonsynonymous SNV
Pa1T1	chr1	78098418	ZZZ3	c.A622C	p.S208R	exonic nonsynonymous SNV
Pa1T1	chr1	152732074	KPRP	c.C10A	p.Q4K	exonic nonsynonymous SNV
Pa1T1	chr1	159163777	CADM3	c.A740G	p.H247R	exonic nonsynonymous SNV
Pa1T1	chr1	160252892	PEX19	c.T188C	p.L63P	exonic nonsynonymous SNV
Pa1T1	chr1	185988801	HMCN1	c.G5599T	p.V1867L	exonic nonsynonymous SNV
Pa1T1	chr1	241933936	WDR64	c.G2167T	p.D723Y	exonic nonsynonymous SNV
Pa1T1	chr1	247655049	OR2W5	c.C620A	p.P207H	exonic nonsynonymous SNV
Pa1T1	chr10	93902	TUBB8	c.G430T	p.G144W	exonic nonsynonymous SNV
Pa1T1	chr10	7759605	ITIH2	c.A484G	p.M162V	exonic nonsynonymous SNV
Pa1T1	chr10	50740667	ERCC6	c.A344G	p.N115S	exonic nonsynonymous SNV
Pa1T1	chr10	121609050	MCMBP	c.C613G	p.R205G	exonic nonsynonymous SNV
Pa1T1	chr10	128114473	C10orf90			splicing
Pa1T1	chr11	4703844	OR51E2	c.T98C	p.M33T	exonic nonsynonymous SNV
Pa1T1	chr11	5020571	OR51L1	c.T359C	p.M120T	exonic nonsynonymous SNV
Pa1T1	chr11	10874642	ZBED5	c.G1851C	p.W617C	exonic nonsynonymous SNV
Pa1T1	chr11	20136144	NAV2	c.G7135A	p.G2379R	exonic nonsynonymous SNV
Pa1T1	chr11	32124876	RCN1	c.A738T	p.L246F	exonic nonsynonymous SNV
Pa1T1	chr11	40137665	LRRC4C	c.G178T	p.V60L	exonic nonsynonymous SNV
Pa1T1	chr11	55579416	OR5L1	c.T474G	p.I158M	exonic nonsynonymous SNV
Pa1T1	chr11	59949206	MS4A6A	c.A79T	p.T27S	exonic nonsynonymous SNV
Pa1T1	chr11	99941236	CNTN5	c.G1243T	p.G415X	exonic stopgain SNV
Pa1T1	chr12	14631298	ATF7IP	c.C2989T	p.Q997X	exonic stopgain SNV
Pa1T1	chr12	19671632	AEBP2	c.A1532G	p.N511S	exonic nonsynonymous SNV
Pa1T1	chr12	25398284	KRAS	c.G35C	p.G12A	exonic nonsynonymous SNV
Pa1T1	chr12	47172403	SLC38A4	c.C874T	p.R292C	exonic nonsynonymous SNV
Pa1T1	chr12	49883276	SPATS2	c.A134G	p.N45S	exonic nonsynonymous SNV
Pa1T1	chr12	78401045	NAV3	c.C1727T	p.A576V	exonic nonsynonymous SNV
Pa1T1	chr12	133393192	GOLGA3	c.G340T	p.G114C	exonic nonsynonymous SNV
Pa1T1	chr13	28367759	GSX1	c.A469G	p.T157A	exonic nonsynonymous SNV
Pa1T1	chr13	75884184	TBC1D4	c.G2487C	p.K829N	exonic nonsynonymous SNV
Pa1T1	chr13	114201680	TMCO3	c.G1756C	p.V586L	exonic nonsynonymous SNV
Pa1T1	chr14	20296157	OR4N2	c.G550T	p.V184F	exonic nonsynonymous SNV
Pa1T1	chr14	70634081	SLC8A3	c.C1059A	p.F353L	exonic nonsynonymous SNV
Pa1T1	chr15	23811099	MKRN3	c.C170A	p.P57H	exonic nonsynonymous SNV
Pa1T1	chr15	68657092	ITGA11	c.C310A	p.R104S	exonic nonsynonymous SNV
Pa1T1	chr15	72499184	PKM2	c.G1247T	p.R416L	exonic nonsynonymous SNV
Pa1T1	chr15	79185906	MORF4L1	c.A683G	p.N228S	exonic nonsynonymous SNV
Pa1T1	chr15	99678247	TTC23	c.G1312T	p.G438W	exonic nonsynonymous SNV
Pa1T1	chr16	23641317	PALB2	c.A2158C	p.T720P	exonic nonsynonymous SNV

Pa1T1	chr16	30976229	SETD1A	c.G1166T	p.R389L	exonic nonsynonymous SNV
Pa1T1	chr17	3119101	OR1A1	c.C187A	p.L63I	exonic nonsynonymous SNV
Pa1T1	chr17	7604096	WRAP53	c.A680G	p.Y227C	exonic nonsynonymous SNV
Pa1T1	chr17	7662251	DNAH2	c.T2257C	p.W753R	exonic nonsynonymous SNV
Pa1T1	chr17	7710792	DNAH2	c.A9617G	p.Y3206C	exonic nonsynonymous SNV
Pa1T1	chr17	10354754	MYH4	c.A3754T	p.M1252L	exonic nonsynonymous SNV
Pa1T1	chr17	73753364	ITGB4	c.A5302T	p.T1768S	exonic nonsynonymous SNV
Pa1T1	chr18	9588086	PPP4R1			splicing
Pa1T1	chr18	14752603	ANKRD30B	c.A260T	p.E87V	exonic nonsynonymous SNV
Pa1T1	chr18	29039806	DSG3			splicing
Pa1T1	chr18	72238479	CNDP1	c.C815A	p.P272Q	exonic nonsynonymous SNV
Pa1T1	chr19	1207146	STK11	c.G234T	p.K78N	exonic nonsynonymous SNV
Pa1T1	chr19	52942380	ZNF534	c.G1706T	p.G569V	exonic nonsynonymous SNV
Pa1T1	chr2	44003989	DYNC2LI1	c.G77T	p.G26V	exonic nonsynonymous SNV
Pa1T1	chr2	54876244	SPTBN1	c.G5119A	p.V1707M	exonic nonsynonymous SNV
Pa1T1	chr2	61417498	USP34	c.C9781G	p.L3261V	exonic nonsynonymous SNV
Pa1T1	chr2	189874945	COL3A1	c.G3865T	p.A1289S	exonic nonsynonymous SNV
Pa1T1	chr2	210837902	UNC80	c.G8297T	p.S2766I	exonic nonsynonymous SNV
Pa1T1	chr2	241069366	MYEOV2	c.C343A	p.R115S	exonic nonsynonymous SNV
Pa1T1	chr20	10256199	SNAP25	c.G60T	p.Q20H	exonic nonsynonymous SNV
Pa1T1	chr20	25491311	NINL	c.A564T	p.Q188H	exonic;splicing nonsynonymous SNV
Pa1T1	chr20	49576196	MOCS3	c.G817T	p.G273C	exonic nonsynonymous SNV
Pa1T1	chr20	49576197	MOCS3	c.G818C	p.G273A	exonic nonsynonymous SNV
Pa1T1	chr20	57782030	ZNF831	c.C3946G	p.R1316G	exonic nonsynonymous SNV
Pa1T1	chr21	10942741	TPTE	c.G700T	p.G234X	exonic stopgain SNV
Pa1T1	chr21	35468155	SLC5A3	c.G658T	p.A220S	exonic nonsynonymous SNV
Pa1T1	chr22	40061613	CACNA1I	c.G3962T	p.G1321V	exonic nonsynonymous SNV
Pa1T1	chr3	1371468	CNTN6			splicing
Pa1T1	chr3	14708930	C3orf19			splicing
Pa1T1	chr3	148763879	HLTF	c.C2060T	p.A687V	exonic nonsynonymous SNV
Pa1T1	chr3	172835192	SPATA16	c.G330A	p.M110I	exonic nonsynonymous SNV
Pa1T1	chr4	83905568	LIN54	c.A430G	p.T144A	exonic nonsynonymous SNV
Pa1T1	chr4	96761571	PDHA2	c.C270A	p.H90Q	exonic nonsynonymous SNV
Pa1T1	chr4	110791769	LRIT3	c.G1729T	p.A577S	exonic nonsynonymous SNV
Pa1T1	chr4	126411155	FAT4	c.C13178A	p.S4393X	exonic stopgain SNV
Pa1T1	chr4	138451997	PCDH18	c.T1246A	p.L416I	exonic nonsynonymous SNV
Pa1T1	chr4	162307402	FSTL5	c.G2041T	p.G681C	exonic nonsynonymous SNV
Pa1T1	chr4	164247487	NPY1R	c.G220C	p.V74L	exonic nonsynonymous SNV
Pa1T1	chr4	189068316	TRIML1	c.C1197A	p.H399Q	exonic nonsynonymous SNV
Pa1T1	chr5	819654	ZDHHC11	c.G1132C	p.G378R	exonic nonsynonymous SNV
Pa1T1	chr5	16694607	MYO10	c.G3673T	p.G1225C	exonic nonsynonymous SNV
Pa1T1	chr5	115840579	SEMA6A	c.C62G	p.P21R	exonic nonsynonymous SNV
Pa1T1	chr5	133914716	PHF15	c.A2262T	p.Q754H	exonic nonsynonymous SNV
Pa1T1	chr5	147793813	FBXO38	c.A1208C	p.K403T	exonic nonsynonymous SNV

Pa1T1	chr5	151784447	NMUR2	c.G228A	p.M76I	exonic nonsynonymous SNV
Pa1T1	chr5	173316747	CPEB4	c.A11T	p.Y4F	exonic nonsynonymous SNV
Pa1T1	chr6	112537568	LAMA4			splicing
Pa1T1	chr7	20784934	ABCB5	c.T3302C	p.V1101A	exonic nonsynonymous SNV
Pa1T1	chr7	36656066	AOAH	c.G766A	p.G256R	exonic nonsynonymous SNV
Pa1T1	chr7	103417070	RELN	c.A478T	p.T160S	exonic nonsynonymous SNV
Pa1T1	chr7	142013452	TCRBV7S1A1N2T	c.G307T	p.E103X	exonic stopgain SNV
Pa1T1	chr8	4494955	CSMD1	c.C211G	p.Q71E	exonic nonsynonymous SNV
Pa1T1	chr8	26227709	PPP2R2A	c.G1154T	p.R385L	exonic nonsynonymous SNV
Pa1T1	chr8	42587114	CHRNB3	c.T664A	p.F222I	exonic nonsynonymous SNV
Pa1T1	chr8	48739314	PRKDC	c.G8683C	p.A2895P	exonic nonsynonymous SNV
Pa1T1	chr8	63928010	GGH	c.C838T	p.R280W	exonic nonsynonymous SNV
Pa1T1	chr9	5906928	MLANA	c.G218T	p.R73I	exonic nonsynonymous SNV
Pa1T1	chr9	90501777	C9orf79	c.C2375T	p.A792V	exonic nonsynonymous SNV
Pa1T1	chr9	91616327	S1PR3	c.A212T	p.H71L	exonic nonsynonymous SNV
Pa1T1	chr9	119380614	ASTN2	c.A3191T	p.D1064V	exonic nonsynonymous SNV
Pa1T1	chr9	126219666	DENND1A	c.G1147C	p.D383H	exonic nonsynonymous SNV
Pa1T1	chrX	27766412	DCAF8L2	c.G1400T	p.R467I	exonic nonsynonymous SNV
Pa1T1	chrX	34962701	FAM47B	c.G1753A	p.G585S	exonic nonsynonymous SNV
Pa1T1	chrX	41205854	DDX3X	c.A1594G	p.T532A	exonic nonsynonymous SNV
Pa1T1	chrX	149639392	MAMLD1	c.A1547T	p.K516I	exonic nonsynonymous SNV
Pa1T2	chr1	16260319	SPEN	c.C7584G	p.D2528E	exonic nonsynonymous SNV
Pa1T2	chr1	17296760	CROCC	c.C5464T	p.R1822W	exonic nonsynonymous SNV
Pa1T2	chr1	33956729	ZSCAN20	c.A871T	p.S291C	exonic nonsynonymous SNV
Pa1T2	chr1	34117939	CSMD2	c.G4570T	p.A1524S	exonic nonsynonymous SNV
Pa1T2	chr1	38343889	INPP5B	c.A1648T	p.K550X	exonic stopgain SNV
Pa1T2	chr1	89630496	GBP7	c.G236C	p.W79S	exonic nonsynonymous SNV
Pa1T2	chr1	175334248	TNR	c.G2485T	p.A829S	exonic nonsynonymous SNV
Pa1T2	chr1	227921215	JMJD4	c.A860C	p.H287P	exonic nonsynonymous SNV
Pa1T2	chr1	236972002	MTR			splicing
Pa1T2	chr1	237791268	RYR2	c.G6328A	p.G2110S	exonic nonsynonymous SNV
Pa1T2	chr1	237948178	RYR2	c.G13166T	p.G4389V	exonic nonsynonymous SNV
Pa1T2	chr1	248004852	OR11L1	c.C347A	p.A116D	exonic nonsynonymous SNV
Pa1T2	chr1	248112672	OR2L8	c.G513T	p.R171S	exonic nonsynonymous SNV
Pa1T2	chr1	249212170	PGBD2	c.G1387T	p.V463F	exonic nonsynonymous SNV
Pa1T2	chr10	87373384	GRID1	c.C2381T	p.T794I	exonic nonsynonymous SNV
Pa1T2	chr11	56043322	OR5T1	c.C208A	p.P70T	exonic nonsynonymous SNV
Pa1T2	chr11	56043323	OR5T1	c.C209A	p.P70Q	exonic nonsynonymous SNV
Pa1T2	chr11	56127946	OR8J1	c.C224T	p.T75I	exonic nonsynonymous SNV
Pa1T2	chr11	56409036	OR5AP2	c.C880A	p.L294I	exonic nonsynonymous SNV
Pa1T2	chr11	61106567	DAK	c.G223A	p.V75I	exonic nonsynonymous SNV
Pa1T2	chr11	68549255	CPT1A	c.G1336T	p.G446C	exonic nonsynonymous SNV
Pa1T2	chr11	103158323	DYNC2H1	c.G11105T	p.C3702F	exonic nonsynonymous SNV
Pa1T2	chr11	105795188	GRIA4	c.T1540C	p.S514P	exonic nonsynonymous SNV

Pa1T2	chr11	108547811	DDX10			splicing
Pa1T2	chr12	29469873	FAR2	c.G1055A	p.W352X	exonic stopgain SNV
Pa1T2	chr12	48379316	COL2A1			splicing
Pa1T2	chr12	57435002	MYO1A	c.C1235A	p.T412N	exonic nonsynonymous SNV
Pa1T2	chr12	81111035	MYF5	c.G193T	p.G65C	exonic nonsynonymous SNV
Pa1T2	chr12	82796801	C12orf26	c.G1171T	p.A391S	exonic nonsynonymous SNV
Pa1T2	chr12	99548233	ANKS1B	c.G38T	p.R13L	exonic nonsynonymous SNV
Pa1T2	chr12	105512280	KIAA1033	c.G492T	p.Q164H	exonic nonsynonymous SNV
Pa1T2	chr12	113534733	DTX1	c.G1852T	p.A618S	exonic nonsynonymous SNV
Pa1T2	chr12	122669154	LRRC43	c.C239T	p.T80M	exonic nonsynonymous SNV
Pa1T2	chr13	77764412	MYCBP2	c.G4129T	p.G1377C	exonic nonsynonymous SNV
Pa1T2	chr14	89088948	EML5	c.A5013T	p.K1671N	exonic;splicing nonsynonymous SNV
Pa1T2	chr14	94155068	UNC79	c.A6619T	p.K2207X	exonic stopgain SNV
Pa1T2	chr14	94909498	SERPINA11	c.C982A	p.P328T	exonic nonsynonymous SNV
Pa1T2	chr14	102510222	DYNC1H1	c.A12524T	p.E4175V	exonic nonsynonymous SNV
Pa1T2	chr15	23811790	MKRN3	c.G861T	p.R287S	exonic nonsynonymous SNV
Pa1T2	chr15	26812850	GABRB3	c.G881T	p.R294L	exonic nonsynonymous SNV
Pa1T2	chr15	27777882	GABRG3	c.G1259C	p.C420S	exonic nonsynonymous SNV
Pa1T2	chr15	89401631	ACAN	c.A5815T	p.R1939X	exonic stopgain SNV
Pa1T2	chr16	22134450	VWA3A	c.C1401A	p.N467K	exonic nonsynonymous SNV
Pa1T2	chr16	28187223	XPO6	c.T401C	p.L134S	exonic nonsynonymous SNV
Pa1T2	chr16	50120203	HEATR3	c.G1451T	p.G484V	exonic nonsynonymous SNV
Pa1T2	chr16	58287995	CCDC113	c.G322A	p.V108I	exonic nonsynonymous SNV
Pa1T2	chr16	68289183	PLA2G15			splicing
Pa1T2	chr16	76556039	CNTNAP4	c.G2649T	p.R883S	exonic nonsynonymous SNV
Pa1T2	chr17	37868282	ERBB2	c.A1003T	p.S335C	exonic nonsynonymous SNV
Pa1T2	chr17	65132255	HELZ	c.A3016T	p.K1006X	exonic stopgain SNV
Pa1T2	chr17	66979880	ABCA9	c.G4610T	p.R1537M	exonic nonsynonymous SNV
Pa1T2	chr17	72348984	KIF19	c.C2005A	p.P669T	exonic nonsynonymous SNV
Pa1T2	chr17	72348985	KIF19	c.C2006A	p.P669Q	exonic nonsynonymous SNV
Pa1T2	chr18	10761012	PIEZO2	c.C3272G	p.S1091C	exonic nonsynonymous SNV
Pa1T2	chr18	14851579	ANKRD30B	c.G3279T	p.M1093I	exonic nonsynonymous SNV
Pa1T2	chr18	28926004	DSG1	c.G1943T	p.G648V	exonic nonsynonymous SNV
Pa1T2	chr18	52901791	TCF4	c.G1780T	p.D594Y	exonic nonsynonymous SNV
Pa1T2	chr19	1207021	STK11	c.C109T	p.Q37X	exonic stopgain SNV
Pa1T2	chr19	9087641	MUC16	c.C4174G	p.H1392D	exonic nonsynonymous SNV
Pa1T2	chr19	10610154	KEAP1	c.G556T	p.G186C	exonic nonsynonymous SNV
Pa1T2	chr19	53572039	ZNF160	c.A1748T	p.H583L	exonic nonsynonymous SNV
Pa1T2	chr19	54969281	LENG8			splicing
Pa1T2	chr2	24439031	ITSN2	c.G3877T	p.A1293S	exonic nonsynonymous SNV
Pa1T2	chr2	88367444	SMYD1	c.C61G	p.L21V	exonic nonsynonymous SNV
Pa1T2	chr2	88424072	FABP1	c.G274T	p.V92L	exonic nonsynonymous SNV
Pa1T2	chr2	116598345	DPP10	c.C2214A	p.H738Q	exonic nonsynonymous SNV
Pa1T2	chr2	136872656	CXCR4	c.A854G	p.H285R	exonic nonsynonymous SNV

Pa1T2	chr2	143798084	KYNU	c.G1129T	p.G377C	exonic nonsynonymous SNV
Pa1T2	chr2	179434184	TTN	c.G68971T	p.V22991F	exonic nonsynonymous SNV
Pa1T2	chr2	182542854	NEUROD1	c.C734T	p.P245L	exonic nonsynonymous SNV
Pa1T2	chr2	183821244	NCKAP1	c.A2117T	p.H706L	exonic nonsynonymous SNV
Pa1T2	chr2	219692062	PRKAG3	c.C910T	p.R304W	exonic nonsynonymous SNV
Pa1T2	chr2	225244633	FAM124B	c.G1025T	p.G342V	exonic nonsynonymous SNV
Pa1T2	chr20	2384271	TGM6	c.G1138T	p.G380C	exonic nonsynonymous SNV
Pa1T2	chr20	45630081	EYA2	c.G124T	p.A42S	exonic nonsynonymous SNV
Pa1T2	chr20	57896241	EDN3	c.G535T	p.V179F	exonic nonsynonymous SNV
Pa1T2	chr21	45535738	PWP2	c.G773T	p.R258L	exonic nonsynonymous SNV
Pa1T2	chr3	108344801	DZIP3	c.G566T	p.R189L	exonic nonsynonymous SNV
Pa1T2	chr3	108751589	MORC1	c.A1543G	p.N515D	exonic nonsynonymous SNV
Pa1T2	chr3	126180704	ZXDC	c.G1801T	p.V601L	exonic nonsynonymous SNV
Pa1T2	chr3	128181919	DNAJB8	c.C170A	p.S57Y	exonic nonsynonymous SNV
Pa1T2	chr3	173998878	NLGN1	c.G2257T	p.V753F	exonic nonsynonymous SNV
Pa1T2	chr4	42145690	BEND4	c.C809G	p.A270G	exonic nonsynonymous SNV
Pa1T2	chr4	47667240	CORIN	c.G1398T	p.L466F	exonic nonsynonymous SNV
Pa1T2	chr4	90856295	MMRN1	c.A1464T	p.L488F	exonic nonsynonymous SNV
Pa1T2	chr4	148575262	PRMT10	c.G1786T	p.V596F	exonic nonsynonymous SNV
Pa1T2	chr4	155507202	FGA	c.C1379T	p.S460L	exonic nonsynonymous SNV
Pa1T2	chr4	164394626	TKTL2	c.G261T	p.W87C	exonic nonsynonymous SNV
Pa1T2	chr5	140574262	PCDHB10	c.G2137T	p.V713L	exonic nonsynonymous SNV
Pa1T2	chr5	153381908	FAM114A2	c.G1159T	p.A387S	exonic nonsynonymous SNV
Pa1T2	chr5	160757984	GABRB2	c.A983T	p.Y328F	exonic nonsynonymous SNV
Pa1T2	chr6	24835966	FAM65B	c.G2236T	p.V746F	exonic nonsynonymous SNV
Pa1T2	chr6	28493857	GPX5	c.C67T	p.P23S	exonic nonsynonymous SNV
Pa1T2	chr6	44199754	SLC29A1	c.C1121G	p.S374C	exonic nonsynonymous SNV
Pa1T2	chr6	66053980	EYS	c.C1550A	p.P517H	exonic nonsynonymous SNV
Pa1T2	chr6	70984465	COL9A1	c.G986C	p.G329A	exonic nonsynonymous SNV
Pa1T2	chr6	152763224	SYNE1	c.G4015C	p.E1339Q	exonic nonsynonymous SNV
Pa1T2	chr7	87800877	ADAM22			splicing
Pa1T2	chr7	124503676	POT1	c.G274T	p.E92X	exonic stopgain SNV
Pa1T2	chr7	146829529	CNTNAP2	c.C1276A	p.L426I	exonic nonsynonymous SNV
Pa1T2	chr8	28608217	EXTL3	c.C2594G	p.A865G	exonic nonsynonymous SNV
Pa1T2	chr8	106456549	ZFPM2	c.G241A	p.D81N	exonic nonsynonymous SNV
Pa1T2	chr8	113358385	CSMD3	c.C6383T	p.P2128L	exonic nonsynonymous SNV
Pa1T2	chr9	36147825	GLIPR2	c.A56T	p.E19V	exonic nonsynonymous SNV
Pa1T2	chr9	86356866	GKAP1			splicing
Pa1T2	chr9	109692939	ZNF462	c.G5981T	p.G1994V	exonic nonsynonymous SNV
Pa1T2	chr9	130289544	FAM129B	c.A244T	p.S82C	exonic nonsynonymous SNV
Pa1T2	chrX	35974153	CXorf22	c.C1250T	p.P417L	exonic nonsynonymous SNV
Pa1T2	chrX	75393438	CXorf26	c.T49C	p.S17P	exonic nonsynonymous SNV
Pa1T2	chrX	152807189	ATP2B3	c.G469T	p.G157W	exonic nonsynonymous SNV
Pa1T3	chr1	47101467	ATPAF1	c.A968T	p.Q323L	exonic nonsynonymous SNV

Pa1T3	chr1	62613981	INADL	c.C5297T	p.A1766V	exonic nonsynonymous SNV
Pa1T3	chr1	67147901	SGIP1	c.C1164A	p.F388L	exonic nonsynonymous SNV
Pa1T3	chr1	110740139	SLC6A17	c.T1733C	p.L578P	exonic nonsynonymous SNV
Pa1T3	chr1	120436609	ADAM30	c.G2351T	p.S784I	exonic nonsynonymous SNV
Pa1T3	chr1	145541844	ITGA10	c.C3367A	p.L1123M	exonic nonsynonymous SNV
Pa1T3	chr1	153750685	SLC27A3	c.C1351G	p.L451V	exonic nonsynonymous SNV
Pa1T3	chr1	158300823	CD1B	c.A91T	p.I31F	exonic nonsynonymous SNV
Pa1T3	chr1	160915001	ITLN2	c.T907A	p.Y303N	exonic nonsynonymous SNV
Pa1T3	chr1	176668724	PAPPA2	c.G3235T	p.V1079L	exonic;splicing nonsynonymous SNV
Pa1T3	chr1	183521033	SMG7	c.A3405T	p.Q1135H	exonic nonsynonymous SNV
Pa1T3	chr1	185143530	SWT1	c.G251C	p.R84T	exonic nonsynonymous SNV
Pa1T3	chr1	202917496	ADIPOR1	c.G194T	p.R65L	exonic nonsynonymous SNV
Pa1T3	chr1	214816550	CENPF	c.G4869T	p.K1623N	exonic nonsynonymous SNV
Pa1T3	chr1	220345378	RAB3GAP2	c.G2430C	p.E810D	exonic nonsynonymous SNV
Pa1T3	chr1	228474738	OBSCN	c.A9542T	p.H3181L	exonic nonsynonymous SNV
Pa1T3	chr1	232600702	SIPA1L2	c.G2704C	p.G902R	exonic nonsynonymous SNV
Pa1T3	chr1	237532901	RYR2	c.G377T	p.S126I	exonic nonsynonymous SNV
Pa1T3	chr1	237777676	RYR2	c.G5248A	p.G1750R	exonic nonsynonymous SNV
Pa1T3	chr1	248112881	OR2L8	c.G722T	p.S241I	exonic nonsynonymous SNV
Pa1T3	chr1	248129494	OR2AK2	c.G861T	p.K287N	exonic nonsynonymous SNV
Pa1T3	chr1	249211962	PGBD2	c.G1179C	p.M393I	exonic nonsynonymous SNV
Pa1T3	chr10	17702512	STAM	c.G90T	p.L30F	exonic nonsynonymous SNV
Pa1T3	chr10	26310476	MYO3A	c.A630T	p.R210S	exonic nonsynonymous SNV
Pa1T3	chr10	29843789	SVIL	c.G83T	p.G28V	exonic nonsynonymous SNV
Pa1T3	chr10	48416605	GDF2	c.G89T	p.R30L	exonic nonsynonymous SNV
Pa1T3	chr10	70332471	TET1	c.A376T	p.K126X	exonic stopgain SNV
Pa1T3	chr10	75156969	ANXA7	c.C323T	p.P108L	exonic nonsynonymous SNV
Pa1T3	chr11	9459743	IPO7	c.T2606A	p.F869Y	exonic nonsynonymous SNV
Pa1T3	chr11	61490357	DAGLA	c.G334A	p.A112T	exonic nonsynonymous SNV
Pa1T3	chr11	62848413	SLC22A24	c.C1577A	p.T526N	exonic nonsynonymous SNV
Pa1T3	chr11	63233845	HRASLS5			splicing
Pa1T3	chr11	85622324	CCDC83	c.G673A	p.V225I	exonic;splicing nonsynonymous SNV
Pa1T3	chr11	101375324	TRPC6	c.G376A	p.V126I	exonic nonsynonymous SNV
Pa1T3	chr11	104761194	CASP12	c.C724A	p.H242N	exonic nonsynonymous SNV
Pa1T3	chr11	111724374	ALG9	c.C649G	p.L217V	exonic nonsynonymous SNV
Pa1T3	chr11	120008273	TRIM29	c.C467T	p.A156V	exonic nonsynonymous SNV
Pa1T3	chr11	124740074	ROBO3	c.C780A	p.F260L	exonic nonsynonymous SNV
Pa1T3	chr12	18891383	CAPZA3	c.G181T	p.V61L	exonic nonsynonymous SNV
Pa1T3	chr12	25398284	KRAS	c.G35T	p.G12V	exonic nonsynonymous SNV
Pa1T3	chr12	56143649	GDF11	c.C1207A	p.R403S	exonic nonsynonymous SNV
Pa1T3	chr12	78400259	NAV3	c.C941A	p.T314N	exonic nonsynonymous SNV
Pa1T3	chr12	88547132	TMTC3	c.G254A	p.S85N	exonic nonsynonymous SNV
Pa1T3	chr12	109702966	ACACB	c.C6994A	p.R2332S	exonic nonsynonymous SNV
Pa1T3	chr13	70681402	KLHL1	c.G430C	p.V144L	exonic nonsynonymous SNV

Pa1T3	chr13	73357671	PIBF1	c.G64C	p.D22H	exonic nonsynonymous SNV
Pa1T3	chr14	94594947	IFI27L2	c.G103T	p.A35S	exonic nonsynonymous SNV
Pa1T3	chr15	32930002	ARHGAP11A	c.G3028T	p.G1010X	exonic stopgain SNV
Pa1T3	chr15	89400507	ACAN	c.C4691A	p.T1564N	exonic nonsynonymous SNV
Pa1T3	chr15	101717888	CHSY1	c.G2114C	p.R705P	exonic nonsynonymous SNV
Pa1T3	chr16	2812745	SRRM2	c.G2216A	p.R739K	exonic nonsynonymous SNV
Pa1T3	chr16	2814619	SRRM2	c.G4090A	p.E1364K	exonic nonsynonymous SNV
Pa1T3	chr16	50322157	ADCY7	c.G67A	p.E23K	exonic nonsynonymous SNV
Pa1T3	chr17	1631362	WDR81	c.C3109T	p.P1037S	exonic nonsynonymous SNV
Pa1T3	chr17	53798062	TMEM100	c.G370T	p.A124S	exonic nonsynonymous SNV
Pa1T3	chr18	10759503	PIEZO2	c.G3659A	p.R1220Q	exonic nonsynonymous SNV
Pa1T3	chr18	19444575	MIB1	c.G2969T	p.S990I	exonic nonsynonymous SNV
Pa1T3	chr18	23615849	SS18	c.G919A	p.E307K	exonic nonsynonymous SNV
Pa1T3	chr18	29040826	DSG3	c.G715T	p.G239C	exonic nonsynonymous SNV
Pa1T3	chr18	72247394	CNDP1	c.C1196T	p.S399F	exonic nonsynonymous SNV
Pa1T3	chr19	6227079	MLLT1	c.C455G	p.S152C	exonic nonsynonymous SNV
Pa1T3	chr19	7944266	LOC388499	c.C508A	p.H170N	exonic nonsynonymous SNV
Pa1T3	chr19	9074769	MUC16	c.G12677T	p.G4226V	exonic nonsynonymous SNV
Pa1T3	chr19	16060286	OR10H4	c.G469T	p.G157W	exonic nonsynonymous SNV
Pa1T3	chr19	23928027	ZNF681	c.G325T	p.E109X	exonic stopgain SNV
Pa1T3	chr19	55176622	LILRB4	c.C871G	p.P291A	exonic nonsynonymous SNV
Pa1T3	chr2	60687669	BCL11A	c.G2378T	p.G793V	exonic nonsynonymous SNV
Pa1T3	chr2	149247132	MBD5	c.G3232A	p.G1078S	exonic nonsynonymous SNV
Pa1T3	chr2	167060957	SCN9A	c.C4383G	p.I1461M	exonic nonsynonymous SNV
Pa1T3	chr2	168114871	XIRP2	c.G1914T	p.Q638H	exonic nonsynonymous SNV
Pa1T3	chr2	170127554	LRP2	c.C2180T	p.S727F	exonic nonsynonymous SNV
Pa1T3	chr2	174104161	ZAK	c.G1296T	p.E432D	exonic nonsynonymous SNV
Pa1T3	chr2	211471638	CPS1	c.G2183T	p.S728I	exonic nonsynonymous SNV
Pa1T3	chr2	219301320	VIL1	c.G1942A	p.D648N	exonic nonsynonymous SNV
Pa1T3	chr2	228176522	COL4A3	c.T4949C	p.V1650A	exonic nonsynonymous SNV
Pa1T3	chr20	12989972	SPTLC3	c.A57T	p.K19N	exonic nonsynonymous SNV
Pa1T3	chr20	37005297	LBP	c.T1432C	p.Y478H	exonic nonsynonymous SNV
Pa1T3	chr20	47858623	DDX27	c.G2089T	p.E697X	exonic stopgain SNV
Pa1T3	chr21	45563163	C21orf33	c.G598T	p.E200X	exonic stopgain SNV
Pa1T3	chr22	38328662	MICALL1	c.G2119C	p.E707Q	exonic nonsynonymous SNV
Pa1T3	chr3	27436587	SLC4A7	c.G2723T	p.G908V	exonic nonsynonymous SNV
Pa1T3	chr3	36896885	TRANK1	c.C4196T	p.T1399I	exonic nonsynonymous SNV
Pa1T3	chr3	38763787	SCN10A	c.T3469A	p.F1157I	exonic nonsynonymous SNV
Pa1T3	chr3	41275702	CTNNB1	c.A1597G	p.I533V	exonic nonsynonymous SNV
Pa1T3	chr3	49570413	DAG1	c.G2469C	p.K823N	exonic nonsynonymous SNV
Pa1T3	chr3	56330222	ERC2	c.C899T	p.A300V	exonic nonsynonymous SNV
Pa1T3	chr3	85932447	CADM2	c.G245A	p.R82K	exonic nonsynonymous SNV
Pa1T3	chr3	108163489	MYH15	c.G2713T	p.A905S	exonic nonsynonymous SNV
Pa1T3	chr3	120957897	STXBP5L	c.C1264T	p.P422S	exonic nonsynonymous SNV

Pa1T3	chr3	127292440	TPRA1	c.A882G	p.X294W	exonic stoploss SNV
Pa1T3	chr3	164750393	SI	c.G2653A	p.D885N	exonic nonsynonymous SNV
Pa1T3	chr3	167164223	SERPINI2	c.A1128G	p.I376M	exonic nonsynonymous SNV
Pa1T3	chr4	2702119	FAM193A	c.A3947G	p.K1316R	exonic nonsynonymous SNV
Pa1T3	chr4	100451099	C4orf17	c.T530C	p.L177P	exonic nonsynonymous SNV
Pa1T3	chr4	109002793	LEF1	c.G671A	p.G224E	exonic nonsynonymous SNV
Pa1T3	chr4	114280347	ANK2	c.G10618T	p.E3540X	exonic stopgain SNV
Pa1T3	chr4	152680069	PET112	c.A182T	p.H61L	exonic nonsynonymous SNV
Pa1T3	chr4	155243485	DCHS2	c.C2809T	p.Q937X	exonic stopgain SNV
Pa1T3	chr4	162380463	FSTL5	c.C1617A	p.S539R	exonic nonsynonymous SNV
Pa1T3	chr4	183550009	ODZ3	c.G955A	p.V319M	exonic nonsynonymous SNV
Pa1T3	chr5	139915025	ANKHD1	c.G6929A	p.G2310E	exonic nonsynonymous SNV
Pa1T3	chr5	141050096	ARAP3	c.C2090T	p.P697L	exonic nonsynonymous SNV
Pa1T3	chr5	149449757	CSF1R	c.G1307A	p.G436D	exonic nonsynonymous SNV
Pa1T3	chr6	12125069	HIVEP1	c.G5041A	p.E1681K	exonic nonsynonymous SNV
Pa1T3	chr6	17629449	NUP153	c.C3074G	p.S1025C	exonic nonsynonymous SNV
Pa1T3	chr6	27277501	POM121L2	c.C2449T	p.Q817X	exonic stopgain SNV
Pa1T3	chr6	43111262	PTK7	c.G2179T	p.V727L	exonic nonsynonymous SNV
Pa1T3	chr6	84233869	PRSS35	c.G709T	p.G237C	exonic nonsynonymous SNV
Pa1T3	chr6	84233870	PRSS35	c.G710C	p.G237A	exonic nonsynonymous SNV
Pa1T3	chr6	160169370	WTAP	c.G421A	p.E141K	exonic nonsynonymous SNV
Pa1T3	chr6	160169397	WTAP	c.G448C	p.D150H	exonic nonsynonymous SNV
Pa1T3	chr6	160679750	SLC22A2	c.G40T	p.E14X	exonic stopgain SNV
Pa1T3	chr7	4026954	SDK1;SDK1	c.A2131T	p.N711Y	exonic;splicing nonsynonymous SNV
Pa1T3	chr7	16572129	LRRC72	c.G100T	p.D34Y	exonic nonsynonymous SNV
Pa1T3	chr7	31378309	NEUROD6	c.C574T	p.Q192X	exonic stopgain SNV
Pa1T3	chr7	36917704	ELMO1	c.G1733T	p.R578L	exonic nonsynonymous SNV
Pa1T3	chr7	98877274	MYH16	c.C397A	p.R133S	exonic nonsynonymous SNV
Pa1T3	chr7	103275996	RELN	c.G2341T	p.V781F	exonic nonsynonymous SNV
Pa1T3	chr7	103777262	ORC5	c.A1228C	p.T410P	exonic nonsynonymous SNV
Pa1T3	chr7	138602157	KIAA1549	c.G2215T	p.D739Y	exonic nonsynonymous SNV
Pa1T3	chr7	144098456	NOBOX	c.C527A	p.S176Y	exonic nonsynonymous SNV
Pa1T3	chr7	157387995	PTPRN2	c.C2500A	p.P834T	exonic nonsynonymous SNV
Pa1T3	chr8	30643127	PPP2CB			UTR3
Pa1T3	chr8	128752787	MYC	c.C948G	p.H316Q	exonic nonsynonymous SNV
Pa1T3	chr9	34256916	KIF24	c.G2689C	p.D897H	exonic nonsynonymous SNV
Pa1T3	chr9	38396064	ALDH1B1	c.C319T	p.R107C	exonic nonsynonymous SNV
Pa1T3	chr9	75773624	ANXA1	c.C80T	p.S27L	exonic nonsynonymous SNV
Pa1T3	chr9	118982225	PAPPA	c.G1928C	p.C643S	exonic nonsynonymous SNV
Pa1T3	chrX	34962111	FAM47B	c.G1163T	p.R388L	exonic nonsynonymous SNV
Pa1T3	chrX	37665639	CYBB			splicing
Pa1T3	chrX	41029259	USP9X	c.G2648T	p.G883V	exonic nonsynonymous SNV
Pa1T3	chrX	123184055	STAG2	c.C913T	p.R305X	exonic stopgain SNV
Pa1T3	chrX	151303563	MAGEA10	c.G530T	p.S177I	exonic nonsynonymous SNV

Pa1LN	chr1	15894532	DNAJC16	c.G2209A	p.E737K	exonic nonsynonymous SNV
Pa1LN	chr1	17281861	CROCC	c.C3520T	p.R1174W	exonic nonsynonymous SNV
Pa1LN	chr1	47101467	ATPAF1	c.A968T	p.Q323L	exonic nonsynonymous SNV
Pa1LN	chr1	67147901	SGIP1	c.C1164A	p.F388L	exonic nonsynonymous SNV
Pa1LN	chr1	110740139	SLC6A17	c.T1733C	p.L578P	exonic nonsynonymous SNV
Pa1LN	chr1	153750685	SLC27A3	c.C1351G	p.L451V	exonic nonsynonymous SNV
Pa1LN	chr1	158300823	CD1B	c.A91T	p.I31F	exonic nonsynonymous SNV
Pa1LN	chr1	158517871	OR6Y1	c.G25C	p.D9H	exonic nonsynonymous SNV
Pa1LN	chr1	179966217	CEP350	c.A925G	p.I309V	exonic nonsynonymous SNV
Pa1LN	chr1	214816550	CENPF	c.G4869T	p.K1623N	exonic nonsynonymous SNV
Pa1LN	chr1	220345378	RAB3GAP2	c.G2430C	p.E810D	exonic nonsynonymous SNV
Pa1LN	chr1	232600702	SIPA1L2	c.G2704C	p.G902R	exonic nonsynonymous SNV
Pa1LN	chr1	237532901	RYR2	c.G377T	p.S126I	exonic nonsynonymous SNV
Pa1LN	chr1	237777676	RYR2	c.G5248A	p.G1750R	exonic nonsynonymous SNV
Pa1LN	chr1	248112881	OR2L8	c.G722T	p.S241I	exonic nonsynonymous SNV
Pa1LN	chr1	248129494	OR2AK2	c.G861T	p.K287N	exonic nonsynonymous SNV
Pa1LN	chr1	249211962	PGBD2	c.G1179C	p.M393I	exonic nonsynonymous SNV
Pa1LN	chr10	1125981	WDR37	c.G266C	p.R89P	exonic nonsynonymous SNV
Pa1LN	chr10	14909154	HSPA14	c.G1066C	p.E356Q	exonic nonsynonymous SNV
Pa1LN	chr10	17702512	STAM	c.G90T	p.L30F	exonic nonsynonymous SNV
Pa1LN	chr10	45958779	MARCH8	c.G908T	p.C303F	exonic nonsynonymous SNV
Pa1LN	chr10	48416605	GDF2	c.G89T	p.R30L	exonic nonsynonymous SNV
Pa1LN	chr11	9459743	IPO7	c.T2606A	p.F869Y	exonic nonsynonymous SNV
Pa1LN	chr11	47307028	MADD	c.G2438A	p.S813N	exonic nonsynonymous SNV
Pa1LN	chr11	62848413	SLC22A24	c.C1577A	p.T526N	exonic nonsynonymous SNV
Pa1LN	chr11	63233845	HRASLS5			splicing
Pa1LN	chr12	8376618	FAM90A1	c.G317A	p.R106K	exonic nonsynonymous SNV
Pa1LN	chr12	18891383	CAPZA3	c.G181T	p.V61L	exonic nonsynonymous SNV
Pa1LN	chr12	25398284	KRAS	c.G35T	p.G12V	exonic nonsynonymous SNV
Pa1LN	chr12	49170887	ADCY6	c.C1376T	p.S459L	exonic;splicing nonsynonymous SNV
Pa1LN	chr12	88547132	TMTC3	c.G254A	p.S85N	exonic nonsynonymous SNV
Pa1LN	chr12	101577999	SLC5A8	c.T965A	p.L322H	exonic;splicing nonsynonymous SNV
Pa1LN	chr13	38151936	POSTN			splicing
Pa1LN	chr14	36946255	SFTA3	c.T182G	p.V61G	exonic nonsynonymous SNV
Pa1LN	chr14	77744736	POMT2			splicing
Pa1LN	chr15	32930002	ARHGAP11A	c.G3028T	p.G1010X	exonic stopgain SNV
Pa1LN	chr15	56122116	NEDD4	c.G3939T	p.Q1313H	exonic nonsynonymous SNV
Pa1LN	chr15	101717888	CHSY1	c.G2114C	p.R705P	exonic nonsynonymous SNV
Pa1LN	chr16	22149717	VWA3A	c.G2200T	p.G734X	exonic stopgain SNV
Pa1LN	chr16	67315673	PLEKHG4	c.C938A	p.A313E	exonic nonsynonymous SNV
Pa1LN	chr17	1631362	WDR81	c.C3109T	p.P1037S	exonic nonsynonymous SNV
Pa1LN	chr17	7750928	KDM6B	c.C1322T	p.S441L	exonic nonsynonymous SNV
Pa1LN	chr17	19850736	AKAP10	c.G960A	p.M320I	exonic nonsynonymous SNV
Pa1LN	chr17	53798062	TMEM100	c.G370T	p.A124S	exonic nonsynonymous SNV

Pa1LN	chr18	10759503	PIEZO2	c.G3659A	p.R1220Q	exonic nonsynonymous SNV
Pa1LN	chr18	45368223	SMAD2	c.C1379G	p.S460X	exonic stopgain SNV
Pa1LN	chr18	48248454	MAPK4	c.G838T	p.E280X	exonic stopgain SNV
Pa1LN	chr19	8591695	MYO1F	c.C2599A	p.L867M	exonic nonsynonymous SNV
Pa1LN	chr19	9060990	MUC16	c.C26456A	p.A8819E	exonic nonsynonymous SNV
Pa1LN	chr19	22157611	ZNF208			splicing
Pa1LN	chr19	37241570	ZNF850	c.A372C	p.K124N	exonic nonsynonymous SNV
Pa1LN	chr19	48305387	TPRX1	c.G881A	p.R294Q	exonic nonsynonymous SNV
Pa1LN	chr19	52250066	FPR1	c.C182A	p.T61N	exonic nonsynonymous SNV
Pa1LN	chr19	55176622	LILRB4	c.C871G	p.P291A	exonic nonsynonymous SNV
Pa1LN	chr19	55607648	PPP1R12C	c.C1007T	p.S336F	exonic nonsynonymous SNV
Pa1LN	chr2	25972596	ASXL2	c.G1829T	p.R610I	exonic nonsynonymous SNV
Pa1LN	chr2	32740275	BIRC6	c.C10787G	p.S3596C	exonic nonsynonymous SNV
Pa1LN	chr2	60687669	BCL11A	c.G2378T	p.G793V	exonic nonsynonymous SNV
Pa1LN	chr2	98340508	ZAP70	c.C9A	p.D3E	exonic nonsynonymous SNV
Pa1LN	chr2	133539876	NCKAP5	c.G4508T	p.G1503V	exonic nonsynonymous SNV
Pa1LN	chr2	149247132	MBD5	c.G3232A	p.G1078S	exonic nonsynonymous SNV
Pa1LN	chr2	174104161	ZAK	c.G1296T	p.E432D	exonic nonsynonymous SNV
Pa1LN	chr2	174130783	ZAK	c.G1708C	p.D570H	exonic nonsynonymous SNV
Pa1LN	chr2	228176522	COL4A3	c.T4949C	p.V1650A	exonic nonsynonymous SNV
Pa1LN	chr20	12989972	SPTLC3	c.A57T	p.K19N	exonic nonsynonymous SNV
Pa1LN	chr20	31685550	BPIFB4	c.C1526T	p.S509F	exonic nonsynonymous SNV
Pa1LN	chr20	33516684	GSS	c.G1372C	p.D458H	exonic nonsynonymous SNV
Pa1LN	chr20	57290287	NPEPL1	c.G1477A	p.D493N	exonic nonsynonymous SNV
Pa1LN	chr20	57768479	ZNF831	c.C2405A	p.A802D	exonic nonsynonymous SNV
Pa1LN	chr20	57768499	ZNF831	c.C2425A	p.P809T	exonic nonsynonymous SNV
Pa1LN	chr21	45563163	C21orf33	c.G598T	p.E200X	exonic stopgain SNV
Pa1LN	chr22	39440148	APOBEC3F	c.G232T	p.A78S	exonic nonsynonymous SNV
Pa1LN	chr3	56330222	ERC2	c.C899T	p.A300V	exonic nonsynonymous SNV
Pa1LN	chr3	57132007	IL17RD	c.G1724T	p.R575L	exonic nonsynonymous SNV
Pa1LN	chr3	108163489	MYH15	c.G2713T	p.A905S	exonic nonsynonymous SNV
Pa1LN	chr3	120957897	STXBP5L	c.C1264T	p.P422S	exonic nonsynonymous SNV
Pa1LN	chr3	127292440	TPRA1	c.A882G	p.X294W	exonic stoploss SNV
Pa1LN	chr3	130114275	COL6A5	c.A3535G	p.I1179V	exonic nonsynonymous SNV
Pa1LN	chr3	164750393	SI	c.G2653A	p.D885N	exonic nonsynonymous SNV
Pa1LN	chr3	175184832	NAALADL2	c.A1393G	p.S465G	exonic nonsynonymous SNV
Pa1LN	chr4	3189477	HTT	c.C5089T	p.Q1697X	exonic stopgain SNV
Pa1LN	chr4	25678062	SLC34A2	c.G1764T	p.Q588H	exonic nonsynonymous SNV
Pa1LN	chr4	27024241	STIM2	c.C1888G	p.P630A	exonic nonsynonymous SNV
Pa1LN	chr4	92519897	FAM190A	c.G2392A	p.E798K	exonic nonsynonymous SNV
Pa1LN	chr4	100451099	C4orf17	c.T530C	p.L177P	exonic nonsynonymous SNV
Pa1LN	chr4	109002793	LEF1	c.G671A	p.G224E	exonic nonsynonymous SNV
Pa1LN	chr4	144468027	SMARCA5	c.A2619T	p.E873D	exonic nonsynonymous SNV
Pa1LN	chr4	162380463	FSTL5	c.C1617A	p.S539R	exonic nonsynonymous SNV

Pa1LN	chr4	167656097	SPOCK3	c.G1313T	p.G438V	exonic nonsynonymous SNV
Pa1LN	chr5	132431911	HSPA4	c.G1772C	p.R591T	exonic nonsynonymous SNV
Pa1LN	chr5	140255862	PCDHA12	c.C805G	p.P269A	exonic nonsynonymous SNV
Pa1LN	chr5	140768847	PCDHGB4	c.G1396T	p.G466X	exonic stopgain SNV
Pa1LN	chr5	141050096	ARAP3	c.C2090T	p.P697L	exonic nonsynonymous SNV
Pa1LN	chr5	145598642	RBM27	c.C154T	p.Q52X	exonic stopgain SNV
Pa1LN	chr5	149449757	CSF1R	c.G1307A	p.G436D	exonic nonsynonymous SNV
Pa1LN	chr6	12296207	EDN1	c.G546C	p.M182I	exonic nonsynonymous SNV
Pa1LN	chr6	84233869	PRSS35	c.G709T	p.G237C	exonic nonsynonymous SNV
Pa1LN	chr6	87970318	ZNF292	c.G6971A	p.G2324E	exonic nonsynonymous SNV
Pa1LN	chr6	160169370	WTAP	c.G421A	p.E141K	exonic nonsynonymous SNV
Pa1LN	chr7	29983678	SCRN1	c.C519G	p.F173L	exonic nonsynonymous SNV
Pa1LN	chr7	31378309	NEUROD6	c.C574T	p.Q192X	exonic stopgain SNV
Pa1LN	chr7	36917704	ELMO1	c.G1733T	p.R578L	exonic nonsynonymous SNV
Pa1LN	chr7	43827562	BLVRA	c.G72A	p.M24I	exonic nonsynonymous SNV
Pa1LN	chr7	103275996	RELN	c.G2341T	p.V781F	exonic nonsynonymous SNV
Pa1LN	chr7	138555962	KIAA1549	c.C4492G	p.P1498A	exonic nonsynonymous SNV
Pa1LN	chr8	77764997	ZFHX4	c.A5840T	p.E1947V	exonic nonsynonymous SNV
Pa1LN	chr8	113331091	CSMD3	c.G7335T	p.M2445I	exonic nonsynonymous SNV
Pa1LN	chr8	113694856	CSMD3	c.A2492T	p.H831L	exonic nonsynonymous SNV
Pa1LN	chr8	142204180	DENND3	c.A3445C	p.I1149L	exonic nonsynonymous SNV
Pa1LN	chr9	34257656	KIF24	c.G1949A	p.G650E	exonic nonsynonymous SNV
Pa1LN	chr9	88938671	ZCCHC6	c.A1994C	p.Q665P	exonic nonsynonymous SNV
Pa1LN	chrX	14882843	FANCB	c.C790A	p.Q264K	exonic nonsynonymous SNV
Pa1LN	chrX	34962111	FAM47B	c.G1163T	p.R388L	exonic nonsynonymous SNV
Pa1LN	chrX	37665639	CYBB			splicing
Pa1LN	chrX	99934352	SYTL4	c.C1616A	p.T539K	exonic nonsynonymous SNV
Pa1LN	chrX	123184055	STAG2	c.C913T	p.R305X	exonic stopgain SNV
Pa1LN	chrX	151303563	MAGEA10	c.G530T	p.S177I	exonic nonsynonymous SNV
Pa2T1	chr1	110884104	RBM15	c.C2077T	p.R693C	exonic nonsynonymous SNV
Pa2T1	chr1	202700177	KDM5B	c.G4144T	p.V1382L	exonic nonsynonymous SNV
Pa2T1	chr12	6167020	VWF	c.G1724A	p.R575H	exonic nonsynonymous SNV
Pa2T1	chr13	50465030	CTAGE10P	c.G304T	p.E102X	exonic stopgain SNV
Pa2T1	chr15	78471109	ACSBG1	c.G1537C	p.E513Q	exonic nonsynonymous SNV
Pa2T1	chr16	16200618	ABCC1	c.A2759G	p.Y920C	exonic nonsynonymous SNV
Pa2T1	chr16	68964193	TMCO7	c.T2696C	p.I899T	exonic nonsynonymous SNV
Pa2T1	chr17	8050302	PER1	c.T1648G	p.C550G	exonic nonsynonymous SNV
Pa2T1	chr17	53798323	TMEM100	c.G109A	p.V37I	exonic nonsynonymous SNV
Pa2T1	chr19	19823785	ZNF14	c.C305T	p.P102L	exonic nonsynonymous SNV
Pa2T1	chr2	74129518	ACTG2	c.G158C	p.S53T	exonic nonsynonymous SNV
Pa2T1	chr2	99182198	INPP4A	c.G2263T	p.A755S	exonic nonsynonymous SNV
Pa2T1	chr2	164468013	FIGN	c.A329T	p.Q110L	exonic nonsynonymous SNV
Pa2T1	chr2	220338240	SPEG	c.G4162A	p.E1388K	exonic nonsynonymous SNV
Pa2T1	chr4	119953154	SYNPO2	c.C3224T	p.S1075L	exonic nonsynonymous SNV

Pa2T1	chr5	90159602	GPR98	c.C17784G	p.I5928M	exonic nonsynonymous SNV
Pa2T1	chr7	24681435	MPP6	c.T218C	p.V73A	exonic nonsynonymous SNV
Pa2T1	chr8	43155692	POTEA	c.A620T	p.H207L	exonic nonsynonymous SNV
Pa2T1	chr8	113256778	CSMD3	c.C10247A	p.T3416N	exonic nonsynonymous SNV
Pa2T2	chr1	92648104	KIAA1107	c.G3550A	p.E1184K	exonic nonsynonymous SNV
Pa2T2	chr1	154998876	DCST2	c.G1513T	p.V505F	exonic nonsynonymous SNV
Pa2T2	chr1	156707303	MRPL24	c.G538A	p.E180K	exonic nonsynonymous SNV
Pa2T2	chr1	172411668	PIGC	c.G95A	p.R32Q	exonic nonsynonymous SNV
Pa2T2	chr10	93593751	TNKS2	c.G1417A	p.G473R	exonic nonsynonymous SNV
Pa2T2	chr10	102296300	HIF1AN	c.G310A	p.D104N	exonic nonsynonymous SNV
Pa2T2	chr11	17632944	OTOG	c.G3151A	p.E1051K	exonic nonsynonymous SNV
Pa2T2	chr11	31484742	IMMP1L	c.G82C	p.E28Q	exonic nonsynonymous SNV
Pa2T2	chr11	66240883	PELI3	c.G628A	p.A210T	exonic nonsynonymous SNV
Pa2T2	chr11	93526947	MED17	c.G691A	p.D231N	exonic nonsynonymous SNV
Pa2T2	chr12	6344675	CD9	c.C481T	p.Q161X	exonic stopgain SNV
Pa2T2	chr12	25398284	KRAS	c.G35T	p.G12V	exonic nonsynonymous SNV
Pa2T2	chr12	46322370	SCAF11	c.C1336T	p.P446S	exonic nonsynonymous SNV
Pa2T2	chr12	49724501	TROAP	c.A1873T	p.S625C	exonic nonsynonymous SNV
Pa2T2	chr12	112610645	C12orf51	c.G11213A	p.R3738Q	exonic nonsynonymous SNV
Pa2T2	chr14	35005459	EAPP	c.C97G	p.L33V	exonic nonsynonymous SNV
Pa2T2	chr14	71063345	MED6	c.G257T	p.R86L	exonic nonsynonymous SNV
Pa2T2	chr14	94048606	UNC79	c.G2188A	p.G730S	exonic nonsynonymous SNV
Pa2T2	chr14	94060167	UNC79	c.G2643T	p.K881N	exonic nonsynonymous SNV
Pa2T2	chr15	24923071	C15orf2	c.C2057T	p.S686F	exonic nonsynonymous SNV
Pa2T2	chr16	27509141	GTF3C1	c.G2167T	p.V723F	exonic;splicing nonsynonymous SNV
Pa2T2	chr16	71318134	FTSJD1	c.G1690T	p.E564X	exonic stopgain SNV
Pa2T2	chr16	72045994	DHODH	c.C67G	p.L23V	exonic nonsynonymous SNV
Pa2T2	chr17	4082260	ANKFY1	c.G2616C	p.L872F	exonic nonsynonymous SNV
Pa2T2	chr17	38948682	KRT28	c.C1392G	p.F464L	exonic nonsynonymous SNV
Pa2T2	chr17	75483634	SPET9	c.G1042T	p.D348Y	exonic;splicing nonsynonymous SNV
Pa2T2	chr18	61306997	SERPINB4	c.C483A	p.N161K	exonic nonsynonymous SNV
Pa2T2	chr19	14883175	EMR2	c.G334C	p.E112Q	exonic nonsynonymous SNV
Pa2T2	chr19	50435687	ATF5	c.T187G	p.F63V	exonic nonsynonymous SNV
Pa2T2	chr19	52709237	PPP2R1A	c.A311T	p.E104V	exonic nonsynonymous SNV
Pa2T2	chr19	56407347	NLRP13	c.G3096C	p.L1032F	exonic nonsynonymous SNV
Pa2T2	chr2	53921062	ASB3	c.C1442G	p.S481C	exonic nonsynonymous SNV
Pa2T2	chr2	135711577	CCNT2	c.G1552A	p.A518T	exonic nonsynonymous SNV
Pa2T2	chr2	141283859	LRP1B	c.C7823A	p.A2608E	exonic nonsynonymous SNV
Pa2T2	chr2	170550810	C2orf77	c.C28T	p.R10W	exonic nonsynonymous SNV
Pa2T2	chr2	179455476	TTN	c.G53272A	p.A17758T	exonic nonsynonymous SNV
Pa2T2	chr20	45003925	ELMO2	c.G1051A	p.E351K	exonic nonsynonymous SNV
Pa2T2	chr20	61538501	DIDO1	c.C1372T	p.Q458X	exonic stopgain SNV
Pa2T2	chr21	39772519	ERG	c.C743A	p.T248K	exonic nonsynonymous SNV
Pa2T2	chr21	41137573	IGSF5	c.G212T	p.W71L	exonic nonsynonymous SNV

Pa2T2	chr22	38328870	MICALL1	c.C2209T	p.R737W	exonic nonsynonymous SNV
Pa2T2	chr3	14703039	C3orf19	c.G310A	p.E104K	exonic nonsynonymous SNV
Pa2T2	chr3	49758728	RNF123	c.C3935T	p.S1312L	exonic nonsynonymous SNV
Pa2T2	chr3	98240100	CLDND1	c.G238A	p.D80N	exonic nonsynonymous SNV
Pa2T2	chr4	144532626	FREM3	c.G5834A	p.R1945H	exonic nonsynonymous SNV
Pa2T2	chr5	41051079	HEATR7B2	c.G1344T	p.Q448H	exonic;splicing nonsynonymous SNV
Pa2T2	chr5	77461456	AP3B1	c.C1208T	p.S403L	exonic nonsynonymous SNV
Pa2T2	chr5	140752108	PCDHGB3	c.G2147A	p.R716Q	exonic nonsynonymous SNV
Pa2T2	chr5	149323933	PDE6A	c.C304T	p.R102C	exonic nonsynonymous SNV
Pa2T2	chr6	50011361	DEFB112	c.C269T	p.T90M	exonic nonsynonymous SNV
Pa2T2	chr7	82784423	PCLO	c.C1534G	p.Q512E	exonic nonsynonymous SNV
Pa2T2	chr8	24259598	ADAMDEC1	c.C1313T	p.S438F	exonic nonsynonymous SNV
Pa2T2	chr8	39624564	ADAM2			splicing
Pa2T2	chr8	72987563	TRPA1	c.G82T	p.D28Y	exonic nonsynonymous SNV
Pa2T2	chr8	121293268	COL14A1	c.A3794G	p.H1265R	exonic nonsynonymous SNV
Pa2T2	chr8	135621053	ZFAT	c.C704T	p.S235L	exonic nonsynonymous SNV
Pa2T2	chr9	131503085	ZER1	c.C1819T	p.Q607X	exonic stopgain SNV
Pa2T2	chrX	55650940	FOXR2	c.C796T	p.R266C	exonic nonsynonymous SNV
Pa2T2	chrX	84363321	SATL1	c.G654A	p.M218I	exonic nonsynonymous SNV
Pa3T1	chr1	7895955	PER3	c.G3348A	p.M1116I	exonic nonsynonymous SNV
Pa3T1	chr1	16065864	SLC25A34	c.G878A	p.R293Q	exonic nonsynonymous SNV
Pa3T1	chr11	124857104	CCDC15	c.A982T	p.I328F	exonic nonsynonymous SNV
Pa3T1	chr12	18891968	CAPZA3	c.C766A	p.L256I	exonic nonsynonymous SNV
Pa3T1	chr12	60173329	SLC16A7	c.A1306G	p.R436G	exonic nonsynonymous SNV
Pa3T1	chr12	78334180	NAV3	c.G325C	p.D109H	exonic nonsynonymous SNV
Pa3T1	chr14	42356681	LRFN5	c.G853A	p.E285K	exonic nonsynonymous SNV
Pa3T1	chr14	55241794	SAMD4A	c.G631A	p.V211I	exonic nonsynonymous SNV
Pa3T1	chr14	99723916	BCL11B	c.A319T	p.R107W	exonic nonsynonymous SNV
Pa3T1	chr16	24802531	TNRC6A	c.C2568A	p.N856K	exonic nonsynonymous SNV
Pa3T1	chr16	66847572	NAE1	c.C662T	p.A221V	exonic nonsynonymous SNV
Pa3T1	chr16	69370408	COG8	c.G585T	p.Q195H	exonic;splicing nonsynonymous SNV
Pa3T1	chr20	32228239	CBFA2T2	c.A1417G	p.T473A	exonic nonsynonymous SNV
Pa3T1	chr20	61542726	DIDO1	c.T239G	p.I80S	exonic nonsynonymous SNV
Pa3T1	chr21	31812674	KRTAP15-1	c.T29A	p.F10Y	exonic nonsynonymous SNV
Pa3T1	chr21	41550926	DSCAM	c.C2875T	p.R959C	exonic nonsynonymous SNV
Pa3T1	chr21	45948399	TSPEAR	c.G858C	p.W286C	exonic nonsynonymous SNV
Pa3T1	chr3	194118527	GP5	c.C485G	p.P162R	exonic nonsynonymous SNV
Pa3T1	chr5	63256489	HTR1A	c.C1058T	p.T353I	exonic nonsynonymous SNV
Pa3T1	chr6	26156823	HIST1H1E	c.G205A	p.A69T	exonic nonsynonymous SNV
Pa3T1	chr8	32600233	NRG1	c.G1216T	p.V406L	exonic nonsynonymous SNV
Pa3T2	chr1	46726221	RAD54L	c.C415A	p.L139I	exonic nonsynonymous SNV
Pa3T2	chr1	63870139	ALG6	c.T273G	p.N91K	exonic nonsynonymous SNV
Pa3T2	chr1	158390470	OR10K2	c.C187A	p.L63I	exonic nonsynonymous SNV
Pa3T2	chr10	32635803	EPC1	c.C41T	p.S14L	exonic nonsynonymous SNV

Pa3T2	chr10	61029689	FAM13C	c.C839T	p.P280L	exonic nonsynonymous SNV
Pa3T2	chr10	105819405	COL17A1	c.G1213T	p.E405X	exonic stopgain SNV
Pa3T2	chr11	93460189	KIAA1731	c.G6547T	p.E2183X	exonic stopgain SNV
Pa3T2	chr11	103104834	DYNC2H1	c.C9512T	p.A3171V	exonic nonsynonymous SNV
Pa3T2	chr11	121016319	TECTA	c.T3599C	p.I1200T	exonic nonsynonymous SNV
Pa3T2	chr11	129784874	PRDM10	c.T2308G	p.F770V	exonic nonsynonymous SNV
Pa3T2	chr12	62687976	USP15	c.C106T	p.R36C	exonic nonsynonymous SNV
Pa3T2	chr12	102158734	GNPTAB	c.G1961C	p.S654T	exonic nonsynonymous SNV
Pa3T2	chr12	105504992	KIAA1033	c.G151A	p.D51N	exonic nonsynonymous SNV
Pa3T2	chr12	105504993	KIAA1033	c.A152T	p.D51V	exonic nonsynonymous SNV
Pa3T2	chr13	103054018	FGF14	c.C11A	p.P4Q	exonic nonsynonymous SNV
Pa3T2	chr18	63477167	CDH7	c.C438G	p.I146M	exonic nonsynonymous SNV
Pa3T2	chr19	13192595	NFIX	c.C1204T	p.H402Y	exonic nonsynonymous SNV
Pa3T2	chr19	22271499	ZNF257	c.A947T	p.Y316F	exonic nonsynonymous SNV
Pa3T2	chr2	207431946	ADAM23			splicing
Pa3T2	chr2	216271105	FN1	c.G2842A	p.G948S	exonic nonsynonymous SNV
Pa3T2	chr20	56188268	ZBP1	c.G621C	p.Q207H	exonic nonsynonymous SNV
Pa3T2	chr22	26231368	MYO18B	c.C3166A	p.R1056S	exonic nonsynonymous SNV
Pa3T2	chr3	167249011	WDR49	c.G1246A	p.A416T	exonic nonsynonymous SNV
Pa3T2	chr3	186953680	MASP1	c.G1979T	p.C660F	exonic nonsynonymous SNV
Pa3T2	chr3	187386991	SST	c.A213C	p.E71D	exonic nonsynonymous SNV
Pa3T2	chr5	32088152	PDZD2	c.A4598G	p.D1533G	exonic nonsynonymous SNV
Pa3T2	chr6	42573479	UBR2	c.A683G	p.Y228C	exonic nonsynonymous SNV
Pa3T2	chr6	90424364	MDN1	c.A6967T	p.N2323Y	exonic nonsynonymous SNV
Pa3T2	chr7	143047525	CLCN1	c.G2464T	p.D822Y	exonic nonsynonymous SNV
Pa3T2	chr9	114982572	PTBP3	c.C1524A	p.H508Q	exonic nonsynonymous SNV
Pa3T2	chr9	130471915	C9orf117	c.G376T	p.V126L	exonic nonsynonymous SNV
Pa3T2	chrX	73812167	RLIM	c.T983G	p.V328G	exonic nonsynonymous SNV
Pa3T2	chrX	142717031	SLITRK4	c.G1894C	p.V632L	exonic nonsynonymous SNV
Pa4T1	chr10	21804594	C10orf140	c.A2158C	p.S720R	exonic nonsynonymous SNV
Pa4T1	chr11	16133439	SOX6	c.C847A	p.P283T	exonic nonsynonymous SNV
Pa4T1	chr11	55563613	OR5D14	c.A582G	p.I194M	exonic nonsynonymous SNV
Pa4T1	chr12	70732604	CNOT2			splicing
Pa4T1	chr13	47469985	HTR2A	c.G57A	p.M19I	exonic nonsynonymous SNV
Pa4T1	chr14	94423217	ASB2	c.G62C	p.R21T	exonic;splicing nonsynonymous SNV
Pa4T1	chr15	55976015	PRTG	c.A512T	p.N171I	exonic nonsynonymous SNV
Pa4T1	chr15	65113196	PIF1	c.C1178A	p.A393D	exonic nonsynonymous SNV
Pa4T1	chr16	82033225	SDR42E1	c.G673T	p.V225L	exonic nonsynonymous SNV
Pa4T1	chr22	38219618	GALR3	c.C205A	p.L69I	exonic nonsynonymous SNV
Pa4T1	chr5	79616527	SPZ1	c.G493A	p.D165N	exonic nonsynonymous SNV
Pa4T1	chr7	55259515	EGFR	c.T2573G	p.L858R	exonic nonsynonymous SNV
Pa4T1	chr9	111938930	EPB41L4B	c.C2534A	p.S845Y	exonic nonsynonymous SNV
Pa4T2	chr1	237838111	RYR2	c.A8795G	p.Y2932C	exonic nonsynonymous SNV
Pa4T2	chr10	125539747	CPXM2	c.G914A	p.R305Q	exonic nonsynonymous SNV

Pa4T2	chr11	71718366	NUMA1	c.A5332C	p.I1778L	exonic nonsynonymous SNV
Pa4T2	chr15	84553880	ADAMTSL3	c.G988A	p.V330M	exonic nonsynonymous SNV
Pa4T2	chr16	3293877	MEFV	c.G1775T	p.G592V	exonic nonsynonymous SNV
Pa4T2	chr16	47156669	NETO2	c.G553A	p.D185N	exonic nonsynonymous SNV
Pa4T2	chr17	48696305	CACNA1G	c.C5717A	p.P1906H	exonic nonsynonymous SNV
Pa4T2	chr19	2852587	ZNF555	c.A524T	p.Q175L	exonic nonsynonymous SNV
Pa4T2	chr19	9639651	ZNF426	c.C1070T	p.S357F	exonic nonsynonymous SNV
Pa4T2	chr19	41763491	AXL	c.C2290T	p.Q764X	exonic stopgain SNV
Pa4T2	chr20	9449304	PLCB4	c.A3335T	p.K1112I	exonic nonsynonymous SNV
Pa4T2	chr21	31709441	KRTAP27-1	c.G546T	p.E182D	exonic nonsynonymous SNV
Pa4T2	chr22	41565587	EP300	c.T4253G	p.L1418R	exonic nonsynonymous SNV
Pa4T2	chr5	35753787	SPEF2	c.G3392A	p.R1131Q	exonic nonsynonymous SNV
Pa4T2	chr5	90670989	ARRDC3	c.C620T	p.S207L	exonic nonsynonymous SNV
Pa4T2	chr5	140594666	PCDHB13	c.A971G	p.D324G	exonic nonsynonymous SNV
Pa4T2	chr5	140603261	PCDHB14	c.C184T	p.R62C	exonic nonsynonymous SNV
Pa4T2	chr6	47575664	CD2AP	c.C1532T	p.P511L	exonic;splicing nonsynonymous SNV
Pa4T2	chr6	166780351	BRP44L	c.G104T	p.G35V	exonic nonsynonymous SNV
Pa4T2	chr7	55220295	EGFR	c.A685T	p.S229C	exonic nonsynonymous SNV
Pa4T2	chr7	103126741	RELN	c.G9886A	p.G3296R	exonic nonsynonymous SNV
Pa4T2	chr9	133760927	ABL1	c.C3307T	p.R1103X	exonic stopgain SNV
Pa5T1	chr1	28059167	FAM76A	c.C302T	p.T101M	exonic;splicing nonsynonymous SNV
Pa5T1	chr1	246755163	CNST	c.C299T	p.A100V	exonic nonsynonymous SNV
Pa5T1	chr1	248224256	OR2L3	c.C273G	p.I91M	exonic nonsynonymous SNV
Pa5T1	chr10	24833987	KIAA1217	c.A5289T	p.K1763N	exonic nonsynonymous SNV
Pa5T1	chr11	49056670	LOC283116			splicing
Pa5T1	chr11	55587670	OR5D18	c.T565A	p.S189T	exonic nonsynonymous SNV
Pa5T1	chr13	49281503	CYSLTR2	c.G550A	p.V184I	exonic nonsynonymous SNV
Pa5T1	chr15	63419565	LACTB	c.C629G	p.T210R	exonic nonsynonymous SNV
Pa5T1	chr16	28124238	XPO6	c.G2138A	p.R713Q	exonic nonsynonymous SNV
Pa5T1	chr17	28384777	EFCAB5	c.C2449A	p.L817I	exonic nonsynonymous SNV
Pa5T1	chr18	53302959	TCF4	c.C170T	p.S57L	exonic nonsynonymous SNV
Pa5T1	chr19	22942019	ZNF99	c.A692G	p.Y231C	exonic nonsynonymous SNV
Pa5T1	chr19	47422005	ARHGAP35	c.G73A	p.E25K	exonic nonsynonymous SNV
Pa5T1	chr2	29150435	WDR43	c.G1174C	p.V392L	exonic;splicing nonsynonymous SNV
Pa5T1	chr2	29295753	C2orf71	c.T1375C	p.F459L	exonic nonsynonymous SNV
Pa5T1	chr2	33484664	LTBP1	c.C2405T	p.A802V	exonic nonsynonymous SNV
Pa5T1	chr2	84744973	DNAH6	c.G23A	p.S8N	exonic nonsynonymous SNV
Pa5T1	chr2	109086159	GCC2	c.T374C	p.I125T	exonic nonsynonymous SNV
Pa5T1	chr2	152319794	RIF1	c.G3760A	p.E1254K	exonic nonsynonymous SNV
Pa5T1	chr2	178307179	AGPS	c.A754G	p.T252A	exonic nonsynonymous SNV
Pa5T1	chr20	57828974	ZNF831	c.G4210A	p.E1404K	exonic nonsynonymous SNV
Pa5T1	chr20	58475836	SYCP2	c.C1220T	p.T407M	exonic nonsynonymous SNV
Pa5T1	chr21	33651294	MIS18A	c.G32A	p.R11K	exonic nonsynonymous SNV
Pa5T1	chr21	33651312	MIS18A	c.G14A	p.R5Q	exonic nonsynonymous SNV

Pa5T1	chr4	7716990	SORCS2	c.C2204T	p.S735F	exonic nonsynonymous SNV
Pa5T1	chr5	93076606	POU5F2	c.G664A	p.G222R	exonic nonsynonymous SNV
Pa5T1	chr5	140580677	PCDHB11	c.G1330A	p.V444I	exonic nonsynonymous SNV
Pa5T1	chr5	161580352	GABRG2	c.G1526T	p.W509L	exonic nonsynonymous SNV
Pa5T1	chr6	97597893	MMS22L	c.G3486T	p.Q1162H	exonic nonsynonymous SNV
Pa5T1	chr7	21641171	DNAH11	c.G3583A	p.E1195K	exonic nonsynonymous SNV
Pa5T1	chr7	55259515	EGFR	c.T2573G	p.L858R	exonic nonsynonymous SNV
Pa5T1	chr7	148947874	ZNF212	c.A517G	p.N173D	exonic nonsynonymous SNV
Pa5T1	chr8	21956079	FAM160B2	c.C914T	p.P305L	exonic nonsynonymous SNV
Pa5T1	chr8	23003285	TNFRSF10D	c.A632T	p.H211L	exonic nonsynonymous SNV
Pa5T1	chr8	41906276	KAT6A	c.C220A	p.P74T	exonic nonsynonymous SNV
Pa5T1	chr8	52287176	PXDNL	c.C3673T	p.R1225W	exonic nonsynonymous SNV
Pa5T1	chr8	142188217	DENND3	c.G2518C	p.V840L	exonic nonsynonymous SNV
Pa5T1	chr8	143921878	GML	c.G25C	p.A9P	exonic nonsynonymous SNV
Pa5T2	chr1	26898726	RPS6KA1	c.G1916T	p.G639V	exonic nonsynonymous SNV
Pa5T2	chr1	33058910	ZBTB8A	c.G378T	p.E126D	exonic nonsynonymous SNV
Pa5T2	chr1	248487129	OR2M7	c.G742T	p.V248L	exonic nonsynonymous SNV
Pa5T2	chr10	50172035	WDFY4	c.C8372T	p.T2791I	exonic nonsynonymous SNV
Pa5T2	chr11	64622867	EHD1	c.G1049C	p.G350A	exonic nonsynonymous SNV
Pa5T2	chr12	110781204	ATP2A2	c.G2486T	p.S829I	exonic nonsynonymous SNV
Pa5T2	chr12	117653122	NOS1	c.A4399T	p.S1467C	exonic nonsynonymous SNV
Pa5T2	chr15	65316110	MTFMT	c.A442G	p.S148G	exonic nonsynonymous SNV
Pa5T2	chr15	65871910	C15orf44	c.A1501T	p.T501S	exonic nonsynonymous SNV
Pa5T2	chr17	26911396	SPAG5	c.A2264T	p.E755V	exonic nonsynonymous SNV
Pa5T2	chr2	26671570	CCDC164	c.G1408A	p.A470T	exonic nonsynonymous SNV
Pa5T2	chr2	179640791	TTN	c.G5800T	p.D1934Y	exonic nonsynonymous SNV
Pa5T2	chr2	230654421	TRIP12	c.C4520T	p.P1507L	exonic nonsynonymous SNV
Pa5T2	chr21	40636611	BRWD1	c.A1660G	p.I554V	exonic;splicing nonsynonymous SNV
Pa5T2	chr22	39909966	SMCR7L	c.G1030A	p.A344T	exonic nonsynonymous SNV
Pa5T2	chr3	113442877	NAA50	c.T74C	p.V25A	exonic nonsynonymous SNV
Pa5T2	chr4	6058486	JAKMIP1	c.G1645T	p.D549Y	exonic;splicing nonsynonymous SNV
Pa5T2	chr5	176666811	NSD1	c.A4247C	p.N1416T	exonic nonsynonymous SNV
Pa5T2	chr6	66094301	EYS	c.T1277A	p.F426Y	exonic nonsynonymous SNV
Pa5T2	chr7	55259515	EGFR	c.T2573G	p.L858R	exonic nonsynonymous SNV
Pa5T2	chr8	4494972	CSMD1	c.G194T	p.G65V	exonic nonsynonymous SNV
Pa5T2	chr8	56436454	XKR4	c.G1621A	p.V541M	exonic nonsynonymous SNV
Pa5T2	chr8	132991600	EFR3A	c.C1507A	p.P503T	exonic nonsynonymous SNV
Pa5T2	chrX	47034490	RBM10	c.A770G	p.Q257R	exonic;splicing nonsynonymous SNV
Pa5T3	chr1	15684702	FHAD1	c.G2593A	p.E865K	exonic nonsynonymous SNV
Pa5T3	chr1	38288356	MTF1	c.G1204T	p.E402X	exonic stopgain SNV
Pa5T3	chr1	55545234	USP24	c.G7177A	p.E2393K	exonic nonsynonymous SNV
Pa5T3	chr1	110717418	SLC6A17	c.G589A	p.E197K	exonic nonsynonymous SNV
Pa5T3	chr1	113662100	LRIG2	c.G2926C	p.E976Q	exonic nonsynonymous SNV
Pa5T3	chr1	152057949	TCHHL1	c.G2209C	p.E737Q	exonic nonsynonymous SNV

Pa5T3	chr1	157803024	CD5L	c.C997T	p.H333Y	exonic nonsynonymous SNV
Pa5T3	chr1	159036081	AIM2	c.C435G	p.I145M	exonic nonsynonymous SNV
Pa5T3	chr1	161641295	FCGR2B	c.C247T	p.Q83X	exonic stopgain SNV
Pa5T3	chr1	180068061	CEP350	c.G9130A	p.D3044N	exonic nonsynonymous SNV
Pa5T3	chr1	212798339	FAM71A	c.C120G	p.F40L	exonic nonsynonymous SNV
Pa5T3	chr10	8106058	GATA3	c.T881A	p.M294K	exonic nonsynonymous SNV
Pa5T3	chr10	43289420	BMS1	c.G1210C	p.E404Q	exonic nonsynonymous SNV
Pa5T3	chr10	88419068	OPN4	c.G676A	p.E226K	exonic nonsynonymous SNV
Pa5T3	chr10	101151238	CNNM1	c.G2884C	p.E962Q	exonic nonsynonymous SNV
Pa5T3	chr10	105164807	PDCD11	c.C431T	p.S144L	exonic nonsynonymous SNV
Pa5T3	chr10	127512184	BCCIP	c.C58A	p.P20T	exonic nonsynonymous SNV
Pa5T3	chr11	16823256	PLEKHA7	c.G2266A	p.D756N	exonic nonsynonymous SNV
Pa5T3	chr11	25071587	LUZP2	c.C769T	p.Q257X	exonic stopgain SNV
Pa5T3	chr11	76507270	TSKU	c.G610C	p.D204H	exonic nonsynonymous SNV
Pa5T3	chr11	118380821	MLL	c.G11059A	p.E3687K	exonic nonsynonymous SNV
Pa5T3	chr11	123900738	OR10G8	c.G409A	p.G137R	exonic nonsynonymous SNV
Pa5T3	chr12	49054310	KANSL2	c.G1615A	p.E539K	exonic nonsynonymous SNV
Pa5T3	chr12	57908986	MARS	c.C2258G	p.S753C	exonic nonsynonymous SNV
Pa5T3	chr12	66232336	HMGA2	c.G236C	p.R79T	exonic nonsynonymous SNV
Pa5T3	chr12	70989988	PTPRB	c.G1099T	p.E367X	exonic stopgain SNV
Pa5T3	chr12	81747109	PPFIA2	c.C1783T	p.L595F	exonic nonsynonymous SNV
Pa5T3	chr12	99192759	ANKS1B	c.G3220C	p.E1074Q	exonic nonsynonymous SNV
Pa5T3	chr12	106729430	TCP11L2	c.G786C	p.Q262H	exonic nonsynonymous SNV
Pa5T3	chr12	124106419	EIF2B1	c.G802T	p.E268X	exonic stopgain SNV
Pa5T3	chr12	133295356	PGAM5	c.C728T	p.P243L	exonic nonsynonymous SNV
Pa5T3	chr13	48832951	ITM2B	c.C583T	p.Q195X	exonic stopgain SNV
Pa5T3	chr13	114078627	ADPRHL1	c.G812C	p.R271T	exonic nonsynonymous SNV
Pa5T3	chr14	70235906	SRSF5	c.C304G	p.P102A	exonic nonsynonymous SNV
Pa5T3	chr14	74762613	ABCD4	c.G612C	p.L204F	exonic nonsynonymous SNV
Pa5T3	chr14	89088995	EML5	c.G4966A	p.E1656K	exonic nonsynonymous SNV
Pa5T3	chr15	51750725	DMXL2	c.G6202A	p.E2068K	exonic nonsynonymous SNV
Pa5T3	chr15	51773309	DMXL2	c.G5994A	p.M1998I	exonic nonsynonymous SNV
Pa5T3	chr15	53997284	WDR72	c.G1249C	p.D417H	exonic nonsynonymous SNV
Pa5T3	chr16	855614	PRR25	c.C172T	p.R58W	exonic nonsynonymous SNV
Pa5T3	chr16	9857724	GRIN2A	c.G3677A	p.G1226D	exonic nonsynonymous SNV
Pa5T3	chr16	28922464	RABEP2	c.C931T	p.R311C	exonic nonsynonymous SNV
Pa5T3	chr16	28947913	CD19	c.C1076T	p.S359L	exonic nonsynonymous SNV
Pa5T3	chr16	67916466	EDC4	c.C3411G	p.I1137M	exonic nonsynonymous SNV
Pa5T3	chr17	1635704	WDR81	c.G4243C	p.E1415Q	exonic nonsynonymous SNV
Pa5T3	chr17	4348429	SPNS3	c.G368C	p.G123A	exonic nonsynonymous SNV
Pa5T3	chr17	6683561	FBXO39	c.G374A	p.R125H	exonic nonsynonymous SNV
Pa5T3	chr17	7139506	PHF23	c.C740G	p.S247C	exonic nonsynonymous SNV
Pa5T3	chr17	29214306	ATAD5	c.G4174A	p.D1392N	exonic nonsynonymous SNV
Pa5T3	chr17	35310206	AATF	c.G304T	p.E102X	exonic stopgain SNV

Pa5T3	chr17	74163790	RNF157	c.C385T	p.R129W	exonic nonsynonymous SNV
Pa5T3	chr18	13071123	CEP192	c.C5260G	p.L1754V	exonic nonsynonymous SNV
Pa5T3	chr18	21736244	CABYR	c.C779T	p.S260F	exonic nonsynonymous SNV
Pa5T3	chr18	43702515	HAUS1	c.G559A	p.A187T	exonic nonsynonymous SNV
Pa5T3	chr18	44089680	LOXHD1	c.C5312T	p.P1771L	exonic nonsynonymous SNV
Pa5T3	chr18	47527680	MYO5B	c.C557T	p.S186L	exonic nonsynonymous SNV
Pa5T3	chr18	72229299	CNDP1	c.G484A	p.G162R	exonic nonsynonymous SNV
Pa5T3	chr19	4538062	LRG1	c.C934G	p.R312G	exonic nonsynonymous SNV
Pa5T3	chr19	6833552	VAV1	c.C1624G	p.Q542E	exonic nonsynonymous SNV
Pa5T3	chr19	12781517	WDR83	c.G388T	p.D130Y	exonic nonsynonymous SNV
Pa5T3	chr19	16001168	CYP4F2	c.G601C	p.D201H	exonic nonsynonymous SNV
Pa5T3	chr19	19349067	NCAN	c.G3256A	p.E1086K	exonic nonsynonymous SNV
Pa5T3	chr19	35617915	LGI4	c.C635T	p.S212F	exonic nonsynonymous SNV
Pa5T3	chr19	36517871	CLIP3	c.C383G	p.A128G	exonic nonsynonymous SNV
Pa5T3	chr19	47422005	ARHGAP35	c.G73A	p.E25K	exonic nonsynonymous SNV
Pa5T3	chr19	49469631	FTL	c.C343T	p.H115Y	exonic nonsynonymous SNV
Pa5T3	chr19	58384327	ZNF814	c.G2431A	p.E811K	exonic nonsynonymous SNV
Pa5T3	chr2	21234660	APOB	c.A5080T	p.N1694Y	exonic nonsynonymous SNV
Pa5T3	chr2	37105049	STRN	c.C1308G	p.D436E	exonic nonsynonymous SNV
Pa5T3	chr2	46609575	EPAS1	c.C2299G	p.Q767E	exonic nonsynonymous SNV
Pa5T3	chr2	48027493	MSH6	c.C2371T	p.R791C	exonic nonsynonymous SNV
Pa5T3	chr2	54081517	GPR75	c.C377T	p.S126L	exonic nonsynonymous SNV
Pa5T3	chr2	165946808	SCN3A	c.A5855C	p.N1952T	exonic nonsynonymous SNV
Pa5T3	chr2	179464008	TTN	c.G48808A	p.E16270K	exonic nonsynonymous SNV
Pa5T3	chr2	179483425	TTN	c.G39148A	p.E13050K	exonic nonsynonymous SNV
Pa5T3	chr2	189873767	COL3A1	c.G3643A	p.G1215S	exonic nonsynonymous SNV
Pa5T3	chr2	192246200	MYO1B	c.G1198A	p.E400K	exonic nonsynonymous SNV
Pa5T3	chr2	192265112	MYO1B	c.G2300A	p.R767Q	exonic nonsynonymous SNV
Pa5T3	chr2	203421253	BMPR2	c.G2865C	p.Q955H	exonic;splicing nonsynonymous SNV
Pa5T3	chr20	20491929	RALGAPA2	c.A4897T	p.R1633X	exonic stopgain SNV
Pa5T3	chr20	20491930	RALGAPA2	c.G4896C	p.L1632F	exonic nonsynonymous SNV
Pa5T3	chr20	30734601	TM9SF4	c.G897A	p.M299I	exonic nonsynonymous SNV
Pa5T3	chr21	37747452	MORC3	c.G2678C	p.R893P	exonic nonsynonymous SNV
Pa5T3	chr21	47666279	MCM3AP	c.C4589T	p.S1530L	exonic nonsynonymous SNV
Pa5T3	chr21	47786996	PCNT	c.C3107T	p.T1036I	exonic nonsynonymous SNV
Pa5T3	chr22	21344752	LZTR1	c.C729G	p.F243L	exonic nonsynonymous SNV
Pa5T3	chr22	39710184	RPL3	c.C879G	p.I293M	exonic nonsynonymous SNV
Pa5T3	chr22	40662063	TNRC6B	c.G1829T	p.R610L	exonic nonsynonymous SNV
Pa5T3	chr3	12198339	TIMP4	c.G333C	p.Q111H	exonic nonsynonymous SNV
Pa5T3	chr3	33438622	UBP1	c.G1186A	p.D396N	exonic nonsynonymous SNV
Pa5T3	chr3	51497215	VPRBP	c.G290A	p.R97Q	exonic nonsynonymous SNV
Pa5T3	chr3	100585756	ABI3BP	c.G976A	p.E326K	exonic nonsynonymous SNV
Pa5T3	chr3	155200784	PLCH1	c.C3055G	p.L1019V	exonic nonsynonymous SNV
Pa5T3	chr3	158315974	MLF1	c.C478G	p.Q160E	exonic nonsynonymous SNV

Pa5T3	chr3	160964196	NMD3	c.C1090T	p.H364Y	exonic nonsynonymous SNV
Pa5T3	chr3	170843718	TNIK	c.G1996C	p.E666Q	exonic nonsynonymous SNV
Pa5T3	chr3	178951964	PIK3CA	c.G3019C	p.G1007R	exonic nonsynonymous SNV
Pa5T3	chr3	178952085	PIK3CA	c.A3140G	p.H1047R	exonic nonsynonymous SNV
Pa5T3	chr4	1976689	WHSC1	c.G3472A	p.D1158N	exonic nonsynonymous SNV
Pa5T3	chr4	6863472	KIAA0232	c.C1363G	p.L455V	exonic nonsynonymous SNV
Pa5T3	chr4	57204708	AASDH	c.C3157T	p.Q1053X	exonic stopgain SNV
Pa5T3	chr4	69094992	TMPRSS11B	c.G929A	p.G310E	exonic nonsynonymous SNV
Pa5T3	chr4	177116616	SPATA4	c.G98A	p.R33Q	exonic nonsynonymous SNV
Pa5T3	chr5	56177936	MAP3K1	c.C2909G	p.S970C	exonic nonsynonymous SNV
Pa5T3	chr5	61783685	IPO11	c.C1426T	p.Q476X	exonic stopgain SNV
Pa5T3	chr5	131039864	FNIP1	c.C1010G	p.S337X	exonic stopgain SNV
Pa5T3	chr5	140215701	PCDHA7	c.G1733A	p.R578K	exonic nonsynonymous SNV
Pa5T3	chr5	140263703	PCDHA13	c.G1850A	p.G617D	exonic nonsynonymous SNV
Pa5T3	chr5	180043904	FLT4	c.G3092A	p.R1031Q	exonic nonsynonymous SNV
Pa5T3	chr6	135748314	AHI1	c.G2755A	p.D919N	exonic nonsynonymous SNV
Pa5T3	chr7	2472620	CHST12	c.G346C	p.D116H	exonic nonsynonymous SNV
Pa5T3	chr7	21599329	DNAH11	c.G801C	p.L267F	exonic nonsynonymous SNV
Pa5T3	chr7	23180561	KLHL7	c.C616G	p.Q206E	exonic nonsynonymous SNV
Pa5T3	chr7	99711688	TAF6	c.G316C	p.E106Q	exonic nonsynonymous SNV
Pa5T3	chr7	100679652	MUC17	c.C4955T	p.S1652L	exonic nonsynonymous SNV
Pa5T3	chr7	121652116	PTPRZ1	c.C3016T	p.L1006F	exonic nonsynonymous SNV
Pa5T3	chr7	154684045	DPP6;DPP6	c.T2453G	p.I818S	exonic;splicing nonsynonymous SNV
Pa5T3	chr8	22463618	KIAA1967	c.C79G	p.L27V	exonic nonsynonymous SNV
Pa5T3	chr8	48697723	PRKDC	c.G11055A	p.W3685X	exonic stopgain SNV
Pa5T3	chr8	72987610	TRPA1	c.G35T	p.G12V	exonic nonsynonymous SNV
Pa5T3	chr8	95518878	KIAA1429	c.C3947T	p.S1316F	exonic nonsynonymous SNV
Pa5T3	chr8	105393440	DPYS	c.C1546T	p.Q516X	exonic stopgain SNV
Pa5T3	chr8	144411599	TOP1MT	c.C281G	p.T94S	exonic nonsynonymous SNV
Pa5T3	chr9	35561285	RUSC2	c.G4457A	p.R1486H	exonic nonsynonymous SNV
Pa5T3	chr9	139726759	C9orf86	c.G645A	p.M215I	exonic nonsynonymous SNV
Pa5T3	chrX	47430355	ARAF	c.C1630T	p.R544C	exonic nonsynonymous SNV
Pa5T3	chrX	54028604	PHF8	c.C1233G	p.I411M	exonic nonsynonymous SNV
Pa5T3	chrX	54839925	MAGED2	c.C1213T	p.H405Y	exonic nonsynonymous SNV
Pa5T3	chrX	69549318	KIF4A	c.A842C	p.K281T	exonic nonsynonymous SNV
Pa5T3	chrX	70598223	TAF1	c.G1132A	p.E378K	exonic nonsynonymous SNV
Pa5T3	chrX	105181535	NRK	c.C3760T	p.Q1254X	exonic stopgain SNV
Pa5T3	chrX	114871194	PLS3	c.G795C	p.M265I	exonic nonsynonymous SNV
Pa5T3	chrX	144906471	SLITRK2	c.G2528A	p.S843N	exonic nonsynonymous SNV
Pa5T3	chrX	152936632	PNCK	c.C877T	p.P293S	exonic nonsynonymous SNV
Pa6T1	chr1	89352997	GTF2B	c.T71C	p.V24A	exonic nonsynonymous SNV
Pa6T1	chr11	33596266	C11orf41	c.G3376T	p.V1126F	exonic nonsynonymous SNV
Pa6T1	chr11	123909213	OR10G7	c.C496T	p.P166S	exonic nonsynonymous SNV
Pa6T1	chr12	49360089	WNT10B	c.G959A	p.R320Q	exonic nonsynonymous SNV

Pa6T1	chr13	35632972	NBEA	c.C1211T	p.A404V	exonic nonsynonymous SNV
Pa6T1	chr13	98116677	RAP2A	c.C533T	p.S178F	exonic nonsynonymous SNV
Pa6T1	chr16	9857680	GRIN2A	c.C3721T	p.R1241W	exonic nonsynonymous SNV
Pa6T1	chr17	40970336	BECN1	c.C586G	p.Q196E	exonic nonsynonymous SNV
Pa6T1	chr20	25596742	NANP	c.A566T	p.D189V	exonic nonsynonymous SNV
Pa6T1	chr22	29688537	EWSR1	c.A1121G	p.N374S	exonic nonsynonymous SNV
Pa6T1	chr3	46008691	FYCO1	c.T2135C	p.L712P	exonic nonsynonymous SNV
Pa6T1	chr5	71490283	MAP1B	c.T1152A	p.N384K	exonic nonsynonymous SNV
Pa6T1	chr7	55259515	EGFR	c.T2573G	p.L858R	exonic nonsynonymous SNV
Pa6T2	chr1	32127026	COL16A1	c.C3760A	p.P1254T	exonic nonsynonymous SNV
Pa6T2	chr1	180905321	KIAA1614	c.G2276A	p.G759E	exonic nonsynonymous SNV
Pa6T2	chr12	91501965	LUM	c.C792A	p.N264K	exonic nonsynonymous SNV
Pa6T2	chr17	47246972	B4GALNT2	c.A1583T	p.D528V	exonic nonsynonymous SNV
Pa6T2	chr20	49575825	MOCS3	c.A446G	p.N149S	exonic nonsynonymous SNV
Pa6T2	chr3	100029247	TBC1D23	c.G1414A	p.G472S	exonic;splicing nonsynonymous SNV
Pa6T2	chr3	188327485	LPP	c.C966A	p.H322Q	exonic nonsynonymous SNV
Pa6T2	chr6	37252215	TBC1D22B	c.A776G	p.E259G	exonic nonsynonymous SNV
Pa6T2	chr6	99729251	C6orf168	c.A1019G	p.N340S	exonic nonsynonymous SNV
Pa6T2	chr6	139583765	TXLNB	c.T833C	p.L278P	exonic nonsynonymous SNV
Pa6T2	chr7	55259515	EGFR	c.T2573G	p.L858R	exonic nonsynonymous SNV
Pa6T2	chr7	123334885	WASL	c.A710G	p.D237G	exonic nonsynonymous SNV
Pa6T3	chr1	22083046	USP48	c.A405C	p.E135D	exonic nonsynonymous SNV
Pa6T3	chr1	44134771	KDM4A	c.C1164G	p.S388R	exonic;splicing nonsynonymous SNV
Pa6T3	chr11	14990398	CALCA	c.G373A	p.D125N	exonic nonsynonymous SNV
Pa6T3	chr11	56000129	OR5T2	c.G533T	p.R178I	exonic nonsynonymous SNV
Pa6T3	chr12	46245705	ARID2	c.G3799T	p.E1267X	exonic stopgain SNV
Pa6T3	chr14	55818297	FBXO34	c.G1189T	p.V397L	exonic nonsynonymous SNV
Pa6T3	chr15	44943892	SPG11	c.T1253A	p.I418N	exonic nonsynonymous SNV
Pa6T3	chr16	2126101	TSC2	c.A2672G	p.H891R	exonic nonsynonymous SNV
Pa6T3	chr17	11650994	DNAH9	c.G6521A	p.R2174H	exonic nonsynonymous SNV
Pa6T3	chr18	77470360	CTDP1	c.G787T	p.E263X	exonic stopgain SNV
Pa6T3	chr21	37741449	MORC3	c.G1783T	p.D595Y	exonic nonsynonymous SNV
Pa6T3	chr22	24911170	UPB1	c.C623A	p.S208X	exonic;splicing stopgain SNV
Pa6T3	chr3	47127755	SETD2	c.T5528G	p.L1843R	exonic nonsynonymous SNV
Pa6T3	chr5	149286879	PDE6A	c.G1061A	p.G354D	exonic nonsynonymous SNV
Pa6T3	chr7	55259515	EGFR	c.T2573G	p.L858R	exonic nonsynonymous SNV
Pa6T3	chr8	104928680	RIMS2	c.G1951T	p.G651X	exonic stopgain SNV
Pa6T3	chr8	104928681	RIMS2	c.G1952T	p.G651V	exonic nonsynonymous SNV
Pa6T3	chr8	139736871	COL22A1	c.C2234A	p.P745H	exonic nonsynonymous SNV
Pa6T3	chr9	4860240	RCL1	c.A1087G	p.I363V	exonic nonsynonymous SNV
Pa6T3	chr9	104125156	BAAT	c.C811A	p.H271N	exonic nonsynonymous SNV
Pa6T3	chrX	40556335	MED14	c.G1591T	p.G531X	exonic stopgain SNV

Supplementary Table 5. Shared exonic mutations (excluding mutations in frequently mutated genes in lung adenocarcinomas) among 16 MSLC lesions and 35 independent lung adenocarcinomas*.

	Pa1LN[124]	Pa1T1[121]	Pa1T2[129]	Pa1T3[150]	Pa2T1[24]	Pa2T2[64]	Pa3T1[29]	Pa3T2[40]	Pa4T1[13]	Pa4T2[21]	Pa5T1[41]	Pa5T2[25]	Pa5T3[178]	Pa6T1[12]	Pa6T2[15]	Pa6T3[22]	TCGA-05-5715[130]	TCGA-38-4627[36]	TCGA-44-2659[519]	TCGA-44-2661[75]	TCGA-44-3919[174]	TCGA-44-6145[322]	TCGA-44-6775[80]	TCGA-44-6776[226]	TCGA-49-4487[430]	TCGA-49-6744[286]	TCGA-49-6745[156]	TCGA-49-6761[206]	TCGA-50-5942[81]	TCGA-50-6593[336]	TCGA-50-6673[63]	TCGA-53-7626[397]	TCGA-55-6543[25]	TCGA-55-6712[193]	TCGA-55-6980[19]	TCGA-55-7281[504]	TCGA-55-7574[315]	TCGA-55-7728[106]	TCGA-55-7815[155]	TCGA-67-3771[1407]	TCGA-67-6215[130]	TCGA-67-6216[54]	TCGA-69-7763[89]	TCGA-69-7980[867]	TCGA-73-4676[103]	TCGA-73-7498[136]	TCGA-73-7499[96]	TCGA-75-6205[53]	TCGA-78-7159[343]	TCGA-93-7348[120]	TCGA-95-7948[181]
Pa1LN[124]	124	0	0	57	0	0	0	0	0	0	0	0	0	0	0	0	0	0	0	0	0	0	0	0	0	0	0	0	0	0	0	0	0	0	0	0	0	0	0	0	0	0	0	0	0	0	0	0	0	0	0
Pa1T1[121]	0	121	0	0	0	0	0	0	0	0	0	0	0	0	0	0	0	0	1	0	0	0	0	0	0	0	0	0	0	0	0	0	0	0	0	0	0	0	0	0	0	0	0	0	0	0	0	0	0	0	0
Pa1T2[129]	0	0	129	0	0	0	0	0	0	0	0	0	0	0	0	0	0	0	0	0	0	0	0	0	0	0	0	0	0	0	0	0	0	0	0	0	0	0	0	0	0	0	0	0	0	0	0	0	0	0	0
Pa1T3[150]	57	0	0	150	0	0	0	0	0	0	0	0	0	0	0	0	0	0	0	0	0	0	0	0	0	0	0	0	0	0	0	0	0	0	0	0	0	0	0	0	0	0	0	0	0	0	0	0	0	0	0
Pa2T1[24]	0	0	0	0	24	0	0	0	0	0	0	0	0	0	0	0	0	0	0	0	0	0	0	0	0	0	0	0	0	0	0	0	0	0	0	0	0	0	0	0	0	0	0	0	0	0	0	0	0	0	0
Pa2T2[64]	0	0	0	0	0	64	0	0	0	0	0	0	0	0	0	0	0	0	0	0	0	0	0	0	0	0	0	0	0	0	0	0	0	0	0	0	0	0	0	0	0	0	0	0	0	0	0	0	0	0	0
Pa3T1[29]	0	0	0	0	0	0	29	0	0	0	0	0	0	0	0	0	0	0	0	0	0	0	0	0	0	0	0	0	0	0	0	0	0	0	0	0	0	0	0	0	0	0	0	0	0	0	0	0	0	0	0
Pa3T2[40]	0	0	0	0	0	0	0	40	0	0	0	0	0	0	0	0	0	0	0	0	0	0	0	0	0	0	0	0	0	0	0	0	0	0	0	0	0	0	0	0	0	0	0	0	0	0	0	0	0	0	0
Pa4T1[13]	0	0	0	0	0	0	0	0	13	0	0	0	0	0	0	0	0	0	0	0	0	0	0	0	0	0	0	0	0	0	0	0	0	0	0	0	0	0	0	0	0	0	0	0	0	0	0	0	0	0	0
Pa4T2[21]	0	0	0	0	0	0	0	0	0	21	0	0	0	0	0	0	0	0	0	0	0	0	0	0	0	0	0	0	0	0	0	0	0	0	0	0	0	0	0	0	0	0	0	0	0	0	0	0	0	0	0
Pa5T1[41]	0	0	0	0	0	0	0	0	0	0	41	0	1	0	0	0	0	0	0	0	0	0	0	0	0	0	0	0	0	0	0	0	0	0	0	0	0	0	0	0	0	0	0	0	0	0	0	0	0	0	0
Pa5T2[25]	0	0	0	0	0	0	0	0	0	0	0	25	0	0	0	0	0	0	0	0	0	0	0	0	0	0	0	0	0	0	0	0	0	0	0	0	0	0	0	0	0	0	0	0	0	0	0	0	0	0	0
Pa5T3[178]	0	0	0	0	0	0	0	0	0	0	1	0	178	0	0	0	0	0	0	0	0	0	0	0	0	0	0	0	0	0	0	0	0	0	0	0	0	0	0	0	0	0	0	0	0	0	0	0	0	0	0
Pa6T1[12]	0	0	0	0	0	0	0	0	0	0	0	0	0	12	0	0	0	0	0	0	0	0	0	0	0	0	0	0	0	0	0	0	0	0	0	0	0	0	0	0	0	0	0	0	0	0	0	0	0	0	0
Pa6T2[15]	0	0	0	0	0	0	0	0	0	0	0	0	0	0	15	0	0	0	0	0	0	0	0	0	0	0	0	0	0	0	0	0	0	0	0	0	0	0	0	0	0	0	0	0	0	0	0	0	0	0	0
Pa6T3[22]	0	0	0	0	0	0	0	0	0	0	0	0	0	0	0	22	0	0	0	0	0	0	0	0	0	0	0	0	0	0	0	0	0	0	0	0	0	0	0	0	0	0	0	0	0	0	0	0	0	0	0
TCGA-05-5715[130]	0	0	0	0	0	0	0	0	0	0	0	0	0	0	0	0	130	0	0	0	0	0	0	0	0	0	0	0	0	0	0	0	0	0	0	0	0	0	0	0	0	0	0	0	0	0	0	0	0	0	0
TCGA-38-4627[36]	0	0	0	0	0	0	0	0	0	0	0	0	0	0	0	0	0	36	0	0	0	0	0	0	0	1	0	0	0	0	0	0	0	0	0	0	0	0	0	0	0	0	0	0	0	0	0	0	0	0	0
TCGA-44-2659[519]	0	1	0	0	0	0	0	0	0	0	0	0	0	0	0	0	0	0	519	0	1	1	0	0	0	0	0	0	0	0	0	1	0	0	0	1	0	0	0	1	0	0	0	0	0	0	0	0	0	0	0
TCGA-44-2661[75]	0	0	0	0	0	0	0	0	0	0	0	0	0	0	0	0	0	0	0	75	0	0	0	0	0	1	0	0	0	0	0	0	0	0	0	0	0	0	1	0	0	0	0	0	0	0	0	0	0	0	0
TCGA-44-3919[174]	0	0	0	0	0	0	0	0	0	0	0	0	0	0	0	0	0	0	1	0	174	0	0	0	0	0	0	0	0	0	0	0	0	0	0	0	0	0	0	1	0	0	0	0	0	0	0	0	0	0	0
TCGA-44-6145[322]	0	0	0	0	0	0	0	0	0	0	0	0	0	0	0	0	0	0	1	0	0	322	0	0	0	0	0	0	0	0	0	0	0	0	0	0	0	0	0	0	0	0	0	0	0	0	0	0	0	0	0
TCGA-44-6775[80]	0	0	0	0	0	0	0	0	0	0	0	0	0	0	0	0	0	0	0	0	0	0	80	0	0	0	0	0	0	0	0	0	0	0	0	0	0	0	0	0	0	0	0	0	0	0	0	0	0	0	0
TCGA-44-6776[226]	0	0	0	0	0	0	0	0	0	0	0	0	0	0	0	0	0	0	0	0	0	0	0	226	0	0	0	0	0	0	0	0	0	0	0	0	0	0	0	0	0	0	0	0	0	0	0	0	0	0	1
TCGA-49-4487[430]	0	0	0	0	0	0	0	0	0	0	0	0	0	0	0	0	0	0	0	0	0	0	0	0	430	0	0	0	0	0	0	0	0	0	0	0	0	0	0	0	0	0	0	0	0	0	0	0	0	0	0
TCGA-49-6744[286]	0	0	0	0	0	0	0	0	0	0	0	0	0	0	0	0	0	1	0	1	0	0	0	0	0	286	0	0	0	0	0	0	0	0	0	0	0	0	1	0	0	0	0	0	0	0	0	0	0	0	0
TCGA-49-6745[156]	0	0	0	0	0	0	0	0	0	0	0	0	0	0	0	0	0	0	0	0	0	0	0	0	0	0	156	0	0	0	0	0	0	0	0	0	0	0	0	0	0	0	0	0	0	0	0	0	0	0	0
TCGA-49-6761[206]	0	0	0	0	0	0	0	0	0	0	0	0	0	0	0	0	0	0	0	0	0	0	0	0	0	0	0	206	0	0	0	0	0	0	0	0	0	0	0	0	0	0	0	0	0	0	0	0	0	0	0
TCGA-50-5942[81]	0	0	0	0	0	0	0	0	0	0	0	0	0	0	0	0	0	0	0	0	0	0	0	0	0	0	0	0	81	0	0	0	0	0	0	0	0	0	0	0	0	0	0	0	0	0	0	0	0	0	0
TCGA-50-6593[336]	0	0	0	0	0	0	0	0	0	0	0	0	0	0	0	0	0	0	0	0	0	0	0	0	0	0	0	0	0	336	0	0	0	0	0	0	0	0	0	0	0	0	0	0	0	0	0	0	0	0	0
TCGA-50-6673[63]	0	0	0	0	0	0	0	0	0	0	0	0	0	0	0	0	0	0	0	0	0	0	0	0	0	0	0	0	0	0	63	0	0	0	1	0	0	0	0	0	0	0	0	0	0	0	0	0	1	0	0
TCGA-53-7626[397]	0	0	0	0	0	0	0	0	0	0	0	0	0	0	0	0	0	0	1	0	0	0	0	0	0	0	0	0	0	0	0	397	0	0	0	0	0	0	0	0	0	0	0	0	0	0	0	0	0	0	0
TCGA-55-6543[25]	0	0	0	0	0	0	0	0	0	0	0	0	0	0	0	0	0	0	0	0	0	0	0	0	0	0	0	0	0	0	0	0	25	0	0	0	0	0	0	0	0	0	0	0	0	0	0	0	0	0	0
TCGA-55-6712[193]	0	0	0	0	0	0	0	0	0	0	0	0	0	0	0	0	0	0	0	0	0	0	0	0	0	0	0	0	0	0	0	0	0	193	0	0	0	0	0	0	0	0	0	0	0	0	0	0	0	0	0
TCGA-55-6980[19]	0	0	0	0	0	0	0	0	0	0	0	0	0	0	0	0	0	0	0	0	0	0	0	0	0	0	0	0	0	0	1	0	0	0	19	0	0	0	0	0	0	0	0	0	0	0	0	0	1	0	0
TCGA-55-7281[504]	0	0	0	0	0	0	0	0	0	0	0	0	0	0	0	0	0	0	1	0	0	0	0	0	0	0	0	0	0	0	0	0	0	0	0	504	0	0	0	0	0	0	0	0	0	0	0	0	0	0	0
TCGA-55-7574[315]	0	0	0	0	0	0	0	0	0	0	0	0	0	0	0	0	0	0	0	0	0	0	0	0	0	0	0	0	0	0	0	0	0	0	0	0	315	0	0	0	0	0	0	0	0	0	0	0	0	0	0
TCGA-55-7728[106]	0	0	0	0	0	0	0	0	0	0	0	0	0	0	0	0	0	0	0	0	0	0	0	0	0	0	0	0	0	0	0	0	0	0	0	0	0	106	0	0	0	0	0	1	0	1	0	0	0	0	0
TCGA-55-7815[155]	0	0	0	0	0	0	0	0	0	0	0	0	0	0	0	0	0	0	0	1	0	0	0	0	0	1	0	0	0	0	0	0	0	0	0	0	0	0	155	0	0	0	0	0	0	0	0	0	0	0	0
TCGA-67-3771[1407]	0	0	0	0	0	0	0	0	0	0	0	0	0	0	0	0	0	0	1	0	1	0	0	0	0	0	0	0	0	0	0	0	0	0	0	0	0	0	0	1407	1	0	0	0	0	0	0	0	0	1	0
TCGA-67-6215[130]	0	0	0	0	0	0	0	0	0	0	0	0	0	0	0	0	0	0	0	0	0	0	0	0	0	0	0	0	0	0	0	0	0	0	0	0	0	0	0	1	130	0	0	0	0	0	0	0	0	0	0
TCGA-67-6216[54]	0	0	0	0	0	0	0	0	0	0	0	0	0	0	0	0	0	0	0	0	0	0	0	0	0	0	0	0	0	0	0	0	0	0	0	0	0	0	0	0	0	54	0	0	0	0	0	0	0	0	0
TCGA-69-7763[89]	0	0	0	0	0	0	0	0	0	0	0	0	0	0	0	0	0	0	0	0	0	0	0	0	0	0	0	0	0	0	0	0	0	0	0	0	0	0	0	0	0	0	89	0	0	0	0	0	1	0	0
TCGA-69-7980[867]	0	0	0	0	0	0	0	0	0	0	0	0	0	0	0	0	0	0	0	0	0	0	0	0	0	0	0	0	0	0	0	0	0	0	0	0	0	1	0	0	0	0	0	867	0	1	0	0	0	0	0
TCGA-73-4676[103]	0	0	0	0	0	0	0	0	0	0	0	0	0	0	0	0	0	0	0	0	0	0	0	0	0	0	0	0	0	0	0	0	0	0	0	0	0	0	0	0	0	0	0	0	103	0	0	0	0	0	0
TCGA-73-7498[136]	0	0	0	0	0	0	0	0	0	0	0	0	0	0	0	0	0	0	0	0	0	0	0	0	0	0	0	0	0	0	0	0	0	0	0	0	0	1	0	0	0	0	0	1	0	136	0	0	0	0	0
TCGA-73-7499[96]	0	0	0	0	0	0	0	0	0	0	0	0	0	0	0	0	0	0	0	0	0	0	0	0	0	0	0	0	0	0	0	0	0	0	0	0	0	0	0	0	0	0	0	0	0	0	96	0	0	0	0
TCGA-75-6205[53]	0	0	0	0	0	0	0	0	0	0	0	0	0	0	0	0	0	0	0	0	0	0	0	0	0	0	0	0	0	0	0	0	0	0	0	0	0	0	0	0	0	0	0	0	0	0	0	53	0	0	0
TCGA-78-7159[343]	0	0	0	0	0	0	0	0	0	0	0	0	0	0	0	0	0	0	0	0	0	0	0	0	0	0	0	0	0	0	1	0	0	0	1	0	0	0	0	0	0	0	1	0	0	0	0	0	343	0	0
TCGA-93-7348[120]	0	0	0	0	0	0	0	0	0	0	0	0	0	0	0	0	0	0	0	0	0	0	0	0	0	0	0	0	0	0	0	0	0	0	0	0	0	0	0	1	0	0	0	0	0	0	0	0	0	120	0
TCGA-95-7948[181]	0	0	0	0	0	0	0	0	0	0	0	0	0	0	0	0	0	0	0	0	0	0	0	1	0	0	0	0	0	0	0	0	0	0	0	0	0	0	0	0	0	0	0	0	0	0	0	0	0	0	181

*Mutation data of independent lung adenocarcinomas were obtained from TCGA. To minimize clinical parameters that may impact the tumor mutation burden and subsequently, the probability of having shared common mutations, only never smokers or light smokers with T2a or earlier T stage tumors from the TCGA study were included (N = 35). Differences in platform and exome capture methods were minimized by including only exonic mutations for this analysis. Mutations in 18 frequently mutated genes in lung adenocarcinomas were excluded from this analysis. Number of shared common mutations (identical nucleotide substitutions at the exact same genomic coordinates) between any pair of these 51 tumors are highlighted. In order to maximize the opportunity to uncover evidence of relatedness in MSLC cohort, all validated mutations plus all mutations that were called by both VarScan and MuTect were included for this analysis.

Supplementary Table 6. Shared exonic mutations among 16 MSLC lesions and 35 independent lung adenocarcinomas*.

	Pa1LN[125]	Pa1T1[124]	Pa1T2[130]	Pa1T3[151]	Pa2T1[24]	Pa2T2[65]	Pa3T1[29]	Pa3T2[40]	Pa4T1[14]	Pa4T2[22]	Pa5T1[42]	Pa5T2[26]	Pa5T3[178]	Pa6T1[13]	Pa6T2[16]	Pa6T3[24]	TCGA-05-5715[130]	TCGA-38-4627[38]	TCGA-44-2659[520]	TCGA-44-2661[75]	TCGA-44-3919[174]	TCGA-44-6145[324]	TCGA-44-6775[82]	TCGA-44-6776[229]	TCGA-49-4487[432]	TCGA-49-6744[289]	TCGA-49-6745[156]	TCGA-49-6761[207]	TCGA-50-5942[83]	TCGA-50-6593[336]	TCGA-50-6673[64]	TCGA-53-7626[398]	TCGA-55-6543[26]	TCGA-55-6712[193]	TCGA-55-6980[19]	TCGA-55-7281[507]	TCGA-55-7574[315]	TCGA-55-7728[107]	TCGA-55-7815[157]	TCGA-67-3771[1412]	TCGA-67-6215[131]	TCGA-67-6216[54]	TCGA-69-7763[89]	TCGA-69-7980[872]	TCGA-73-4676[105]	TCGA-73-7498[139]	TCGA-73-7499[96]	TCGA-75-6205[53]	TCGA-78-7159[343]	TCGA-93-7348[120]	TCGA-95-7948[181]
Pa1LN[125]	125	0	0	58	0	1	0	0	0	0	0	0	0	0	0	0	0	0	0	0	0	1	0	0	0	0	0	0	0	0	0	0	0	0	0	0	0	1	1	0	0	0	0	0	0	0	0	0	0	0	0
Pa1T1[124]	0	124	0	0	0	0	0	0	0	0	0	0	0	0	0	0	0	0	1	0	0	0	0	0	0	0	0	0	0	0	0	0	0	0	0	0	0	0	0	0	0	0	0	1	0	0	0	0	0	0	0
Pa1T2[130]	0	0	130	0	0	0	0	0	0	0	0	0	0	0	0	0	0	0	0	0	0	0	0	0	0	0	0	0	0	0	0	0	0	0	0	0	0	0	0	0	0	0	0	0	0	0	0	0	0	0	0
Pa1T3[151]	58	0	0	151	0	1	0	0	0	0	0	0	0	0	0	0	0	0	0	0	0	1	0	0	0	0	0	0	0	0	0	0	0	0	0	0	0	1	1	0	0	0	0	0	0	0	0	0	0	0	0
Pa2T1[24]	0	0	0	0	24	0	0	0	0	0	0	0	0	0	0	0	0	0	0	0	0	0	0	0	0	0	0	0	0	0	0	0	0	0	0	0	0	0	0	0	0	0	0	0	0	0	0	0	0	0	0
Pa2T2[65]	1	0	0	1	0	65	0	0	0	0	0	0	0	0	0	0	0	0	0	0	0	1	0	0	0	0	0	0	0	0	0	0	0	0	0	0	0	1	1	0	0	0	0	0	0	0	0	0	0	0	0
Pa3T1[29]	0	0	0	0	0	0	29	0	0	0	0	0	0	0	0	0	0	0	0	0	0	0	0	0	0	0	0	0	0	0	0	0	0	0	0	0	0	0	0	0	0	0	0	0	0	0	0	0	0	0	0
Pa3T2[40]	0	0	0	0	0	0	0	40	0	0	0	0	0	0	0	0	0	0	0	0	0	0	0	0	0	0	0	0	0	0	0	0	0	0	0	0	0	0	0	0	0	0	0	0	0	0	0	0	0	0	0
Pa4T1[14]	0	0	0	0	0	0	0	0	14	0	1	1	0	1	1	1	0	1	0	0	0	0	0	0	0	0	0	0	0	0	0	0	0	0	0	0	0	0	0	0	0	0	0	0	0	0	0	0	0	0	0
Pa4T2[22]	0	0	0	0	0	0	0	0	0	22	0	0	0	0	0	0	0	0	0	0	0	0	0	0	0	0	0	0	0	0	0	0	0	0	0	0	0	0	0	0	0	0	0	0	0	0	0	0	0	0	0
Pa5T1[42]	0	0	0	0	0	0	0	0	1	0	42	1	1	1	1	1	0	1	0	0	0	0	0	0	0	0	0	0	0	0	0	0	0	0	0	0	0	0	0	0	0	0	0	0	0	0	0	0	0	0	0
Pa5T2[26]	0	0	0	0	0	0	0	0	1	0	1	26	0	1	1	1	0	1	0	0	0	0	0	0	0	0	0	0	0	0	0	0	0	0	0	0	0	0	0	0	0	0	0	0	0	0	0	0	0	0	0
Pa5T3[178]	0	0	0	0	0	0	0	0	0	0	1	0	178	0	0	0	0	0	0	0	0	0	0	0	0	0	0	0	0	0	0	0	0	0	0	0	0	0	0	0	0	0	0	0	0	0	0	0	0	0	0
Pa6T1[13]	0	0	0	0	0	0	0	0	1	0	1	1	0	13	1	1	0	1	0	0	0	0	0	0	0	0	0	0	0	0	0	0	0	0	0	0	0	0	0	0	0	0	0	0	0	0	0	0	0	0	0
Pa6T2[16]	0	0	0	0	0	0	0	0	1	0	1	1	0	1	16	1	0	1	0	0	0	0	0	0	0	0	0	0	0	0	0	0	0	0	0	0	0	0	0	0	0	0	0	0	0	0	0	0	0	0	0
Pa6T3[24]	0	0	0	0	0	0	0	0	1	0	1	1	0	1	1	24	0	1	0	0	0	0	0	0	0	0	0	0	0	0	0	0	0	0	0	0	0	0	0	0	0	0	0	0	0	0	0	0	0	0	0
TCGA-05-5715[130]	0	0	0	0	0	0	0	0	0	0	0	0	0	0	0	0	130	0	0	0	0	0	0	0	0	0	0	0	0	0	0	0	0	0	0	0	0	0	0	0	0	0	0	0	0	0	0	0	0	0	0
TCGA-38-4627[38]	0	0	0	0	0	0	0	0	1	0	1	1	0	1	1	1	0	38	0	0	0	0	0	0	0	1	0	0	0	0	0	0	0	0	0	0	0	0	0	0	0	0	0	0	0	0	0	0	0	0	0
TCGA-44-2659[520]	0	1	0	0	0	0	0	0	0	0	0	0	0	0	0	0	0	0	520	0	1	1	0	0	0	0	0	0	0	0	0	1	0	0	0	1	0	0	0	1	0	0	0	0	0	0	0	0	0	0	0
TCGA-44-2661[75]	0	0	0	0	0	0	0	0	0	0	0	0	0	0	0	0	0	0	0	75	0	0	0	0	0	1	0	0	0	0	0	0	0	0	0	0	0	0	1	0	0	0	0	0	0	0	0	0	0	0	0
TCGA-44-3919[174]	0	0	0	0	0	0	0	0	0	0	0	0	0	0	0	0	0	0	1	0	174	0	0	0	0	0	0	0	0	0	0	0	0	0	0	0	0	0	0	1	0	0	0	0	0	0	0	0	0	0	0
TCGA-44-6145[324]	1	0	0	1	0	1	0	0	0	0	0	0	0	0	0	0	0	0	1	0	0	324	0	0	0	0	0	0	0	0	0	0	0	0	0	0	0	1	1	1	0	0	0	0	0	0	0	0	0	0	0
TCGA-44-6775[82]	0	0	0	0	0	0	0	0	0	0	0	0	0	0	0	0	0	0	0	0	0	0	82	0	0	0	0	0	0	0	0	0	0	0	0	0	0	0	0	0	0	0	0	0	0	0	0	0	0	0	0
TCGA-44-6776[229]	0	0	0	0	0	0	0	0	0	0	0	0	0	0	0	0	0	0	0	0	0	0	0	229	0	0	0	1	0	0	0	0	0	0	0	0	0	0	0	0	0	0	0	0	0	0	0	0	0	0	1
TCGA-49-4487[432]	0	0	0	0	0	0	0	0	0	0	0	0	0	1	0	0	0	0	0	0	0	0	0	0	432	0	0	0	0	0	0	0	0	0	0	0	0	0	0	0	0	0	0	0	0	0	0	0	0	0	0
TCGA-49-6744[289]	0	0	0	0	0	0	0	0	0	0	0	0	0	0	0	0	0	1	0	1	0	0	0	0	0	289	0	0	0	0	0	0	0	0	0	0	0	0	1	0	0	0	0	0	0	0	0	0	0	0	0
TCGA-49-6745[156]	0	0	0	0	0	0	0	0	0	0	0	0	0	0	0	0	0	0	0	0	0	0	0	0	0	0	156	0	0	0	0	0	0	0	0	0	0	0	0	0	0	0	0	0	0	0	0	0	0	0	0
TCGA-49-6761[207]	0	0	0	0	0	0	0	0	0	0	0	0	0	0	0	0	0	0	0	0	0	0	0	1	0	0	0	207	0	0	0	0	0	0	0	0	0	0	0	0	0	0	0	0	0	0	0	0	0	0	0
TCGA-50-5942[83]	0	0	0	0	0	0	0	0	0	0	0	0	0	0	0	0	0	0	0	0	0	0	0	0	0	0	0	0	83	0	0	0	0	0	0	0	0	0	0	0	0	0	0	0	0	0	0	0	0	0	0
TCGA-50-6593[336]	0	0	0	0	0	0	0	0	0	0	0	0	0	0	0	0	0	0	0	0	0	0	0	0	0	0	0	0	0	336	0	0	0	0	0	0	0	0	0	0	0	0	0	0	0	0	0	0	0	0	0
TCGA-50-6673[64]	0	0	0	0	0	0	0	0	0	0	0	0	0	0	0	0	0	0	0	0	0	0	0	0	0	0	0	0	0	0	64	0	0	0	1	0	0	0	0	0	0	0	0	0	0	0	0	0	1	0	0
TCGA-53-7626[398]	0	0	0	0	0	0	0	0	0	0	0	0	0	0	0	0	0	0	1	0	0	0	0	0	0	0	0	0	0	0	0	398	0	0	0	0	0	0	0	0	0	0	0	0	0	0	0	0	0	0	0
TCGA-55-6543[26]	0	0	0	0	0	0	0	0	0	0	0	0	0	0	0	0	0	0	0	0	0	0	0	0	0	0	0	0	0	0	0	0	26	0	0	0	0	0	0	0	0	0	0	0	0	0	0	0	0	0	0
TCGA-55-6712[193]	0	0	0	0	0	0	0	0	0	0	0	0	0	0	0	0	0	0	0	0	0	0	0	0	0	0	0	0	0	0	0	0	0	193	0	0	0	0	0	0	0	0	0	0	0	0	0	0	0	0	0
TCGA-55-6980[19]	0	0	0	0	0	0	0	0	0	0	0	0	0	0	0	0	0	0	0	0	0	0	0	0	0	0	0	0	0	0	1	0	0	0	19	0	0	0	0	0	0	0	0	0	0	0	0	0	1	0	0
TCGA-55-7281[507]	0	0	0	0	0	0	0	0	0	0	0	0	0	0	0	0	0	0	1	0	0	0	0	0	0	0	0	0	0	0	0	0	0	0	0	507	0	0	0	0	0	0	0	0	0	1	0	0	0	0	0
TCGA-55-7574[315]	0	0	0	0	0	0	0	0	0	0	0	0	0	0	0	0	0	0	0	0	0	0	0	0	0	0	0	0	0	0	0	0	0	0	0	0	315	0	0	0	0	0	0	0	0	0	0	0	0	0	0
TCGA-55-7728[107]	1	0	0	1	0	1	0	0	0	0	0	0	0	0	0	0	0	0	0	0	0	1	0	0	0	0	0	0	0	0	0	0	0	0	0	0	0	107	1	0	0	0	0	1	0	1	0	0	0	0	0
TCGA-55-7815[157]	1	0	0	1	0	1	0	0	0	0	0	0	0	0	0	0	0	0	0	1	0	1	0	0	0	1	0	0	0	0	0	0	0	0	0	0	0	1	157	0	0	0	0	0	0	0	0	0	0	0	0
TCGA-67-3771[1412]	0	0	0	0	0	0	0	0	0	0	0	0	0	0	0	0	0	0	1	0	1	1	0	0	0	0	0	0	0	0	0	0	0	0	0	0	0	0	0	1412	1	0	0	0	0	0	0	0	0	1	0
TCGA-67-6215[131]	0	0	0	0	0	0	0	0	0	0	0	0	0	0	0	0	0	0	0	0	0	0	0	0	0	0	0	0	0	0	0	0	0	0	0	0	0	0	0	1	131	0	0	0	0	0	0	0	0	0	0
TCGA-67-6216[54]	0	0	0	0	0	0	0	0	0	0	0	0	0	0	0	0	0	0	0	0	0	0	0	0	0	0	0	0	0	0	0	0	0	0	0	0	0	0	0	0	0	54	0	0	0	0	0	0	0	0	0
TCGA-69-7763[89]	0	0	0	0	0	0	0	0	0	0	0	0	0	0	0	0	0	0	0	0	0	0	0	0	0	0	0	0	0	0	0	0	0	0	0	0	0	0	0	0	0	0	89	0	0	0	0	0	1	0	0
TCGA-69-7980[872]	0	1	0	0	0	0	0	0	0	0	0	0	0	0	0	0	0	0	0	0	0	0	0	0	0	0	0	0	0	0	0	0	0	0	0	0	0	1	0	0	0	0	0	872	0	1	0	0	0	0	0
TCGA-73-4676[105]	0	0	0	0	0	0	0	0	0	0	0	0	0	0	0	0	0	0	0	0	0	0	0	0	0	0	0	0	0	0	0	0	0	0	0	0	0	0	0	0	0	0	0	0	105	0	0	0	0	0	0
TCGA-73-7498[139]	0	0	0	0	0	0	0	0	0	0	0	0	0	0	0	0	0	0	0	0	0	0	0	0	0	0	0	0	0	0	0	0	0	0	0	1	0	1	0	0	0	0	0	1	0	139	0	0	0	0	0
TCGA-73-7499[96]	0	0	0	0	0	0	0	0	0	0	0	0	0	0	0	0	0	0	0	0	0	0	0	0	0	0	0	0	0	0	0	0	0	0	0	0	0	0	0	0	0	0	0	0	0	0	96	0	0	0	0
TCGA-75-6205[53]	0	0	0	0	0	0	0	0	0	0	0	0	0	0	0	0	0	0	0	0	0	0	0	0	0	0	0	0	0	0	0	0	0	0	0	0	0	0	0	0	0	0	0	0	0	0	0	53	0	0	0
TCGA-78-7159[343]	0	0	0	0	0	0	0	0	0	0	0	0	0	0	0	0	0	0	0	0	0	0	0	0	0	0	0	0	0	0	1	0	0	0	1	0	0	0	0	0	0	0	1	0	0	0	0	0	343	0	0
TCGA-93-7348[120]	0	0	0	0	0	0	0	0	0	0	0	0	0	0	0	0	0	0	0	0	0	0	0	0	0	0	0	0	0	0	0	0	0	0	0	0	0	0	0	1	0	0	0	0	0	0	0	0	0	120	0
TCGA-95-7948[181]	0	0	0	0	0	0	0	0	0	0	0	0	0	0	0	0	0	0	0	0	0	0	0	1	0	0	0	0	0	0	0	0	0	0	0	0	0	0	0	0	0	0	0	0	0	0	0	0	0	0	181

*Mutation data of independent lung adenocarcinomas were obtained from TCGA. To minimize clinical parameters that may impact the tumor mutation burden and subsequently, the probability of having shared common mutations, only never smokers or light smokers with T2a or earlier T stage tumors from the TCGA study were included (N = 35). Differences in platform and exome capture methods were minimized by including only exonic mutations for this analysis. Number of shared common mutations (identical nucleotide substitutions at the exact same genomic coordinates) between any pair of these 51 tumors are highlighted. In order to maximize the opportunity to uncover evidence of relatedness in MSLC cohort, all validated mutations plus all mutations that were called by both VarScan and MuTect were included for this analysis.

Supplementary Table 7. Structural variants detected by whole genome sequencing in the 3 tumors and one metastatic lymph node from Patient 1.

left chr	left pososition	left strand	right chr	right position	right strand	Pa1T1	Pa1T2	Pa1T3	Pa1LN	FlankingGenes (10kb)*
chr2	129740493	+	chr2	129785472	+	---	DEL	---	---	---
chr2	13143013	+	chr11	82873136	-	CTX	---	---	---	LOC100506474;PCF11
chr2	14892946	-	chr2	4916888	+	ITX	---	---	---	---
chr2	163565096	+	chr2	163568850	+	---	---	DEL	---	KCNH7
chr2	165608170	+	chr2	165683427	+	---	---	DEL	---	COBLL1
chr2	3999899	+	chr11	125701884	-	CTX	---	---	---	LOC100505964;PATE4
chr2	49064563	+	chr2	49073703	+	---	---	DEL	DEL	---
chr2	52532826	+	chr2	52537731	+	---	DEL	---	---	---
chr2	58281759	+	chr2	58281705	+	---	---	---	INS	VRK2
chr2	61784839	+	chr2	61785357	+	---	---	DEL	---	---
chr3	109469055	-	chr3	84632171	+	ITX	---	---	---	---
chr3	133907002	+	chr3	133910949	+	DEL	---	---	---	RYK
chr3	152718	+	chr3	6088272	+	DEL	---	---	---	---
chr3	174296163	+	chr3	174297810	+	DEL	---	---	---	---
chr3	27040645	+	chr3	27041115	+	DEL	---	---	---	---
chr3	4319032	-	chr3	158261082	+	ITX	---	---	---	RSRC1
chr3	57111373	+	chr3	57082484	+	INS	---	---	---	ARHGEF3;SPATA12
chr3	81231166	+	chr3	116161919	-	ITX	---	---	---	LSAMP
chr7	6445420	+	chr7	6377996	+	INS	---	---	---	RAC1;DAGLB;FAM220A
chr10	105193622	+	chr10	105194556	+	DEL	---	---	---	PDCD11
chr10	11010204	+	chr10	11010346	+	---	---	DEL	DEL	---
chr10	6154687	+	chr10	6041806	+	---	---	---	INS	IL2RA;RBM17
chr10	71314972	+	chr10	71315061	+	---	---	---	DEL	---
chr11	82987348	+	chr2	3988486	+	CTX	---	---	---	CCDC90B
chr15	100952636	+	chr15	100952831	+	DEL	---	---	---	CERS3

ITX: Intra-chromosome translocation; CTX: Inter-chromosomal translocation; INS: Insertion; DEL: Deletion; ---: Not detected.

* FlankingGenes refers to any gene occurring within 10 kb both upstream and downstream from the breakpoints.

Shared common variants are highlighted.
